# *N*-glycans from serum IgG and total serum glycoproteins specific for endometriosis

**DOI:** 10.1038/s41598-023-37421-5

**Published:** 2023-06-28

**Authors:** Zsuzsanna Kovács, Fiona Reidy, Louise Glover, Fionnuala M. McAuliffe, Henning Stockmann, Mark T. Kilbane, Patrick J. Twomey, Maire Peters, Merli Saare, Pauline M. Rudd, Meeme Utt, Mary Wingfield, Andres Salumets, Radka Saldova

**Affiliations:** 1 NIBRT GlycoScience Group, National Institute for Bioprocessing Research and Training, Belfield, Blackrock, Dublin, A94 X099 Co. Dublin Ireland; 2 Merrion Fertility Clinic and National Maternity Hospital, Dublin, Ireland; 3 Obstetrics and Gynaecology, UCD Perinatal Research Centre, School of Medicine, University College Dublin, National Maternity Hospital, Dublin, Ireland; 4 Department of Clinical Chemistry, St. Vincent’s University Hospital, Dublin, Ireland; 5 UCD School of Medicine and Medical Science, University College Dublin, Dublin, Ireland; 6 Department of Obstetrics and Gynaecology, Institute of Clinical Medicine, University of Tartu, Tartu, Estonia; 7 Competence Centre On Health Technologies, Tartu, Estonia; 8 Department of Immunology, Institute of Biomedicine and Translational Medicine, University of Tartu, Tartu, Estonia; 9 Division of Obstetrics and Gynecology, Department of Clinical Science, Intervention and Technology, Karolinska Institutet and Karolinska University Hospital, Stockholm, Sweden; 10 College of Health and Agricultural Science (CHAS), UCD School of Medicine, University College Dublin (UCD), Dublin, D07 A8NN Ireland

**Keywords:** Glycobiology, Glycoproteins, Diagnostic markers, Urogenital reproductive disorders, Blood proteins

## Abstract

Endometriosis is a chronic inflammatory gynaecological disease characterized by the growth of endometrial tissue outside the uterine cavity. There are currently no definitive non-invasive diagnostic tools. Glycosylation is the most common posttranslational modification of proteins and altered glycosylation has been found in many diseases, including chronic inflammatory conditions and cancer. Sialylation and galactosylation on serum IgG have previously been found to be altered in endometriosis and serum sialylation changed after Zoladex (Goserelin Acetate) therapy. Using IgG and whole serum glycoproteins, we investigated *N*-glycosylation in two clinical cohorts of women with and without endometriosis. PNGase F-digested serum samples were fluorescently labelled and *N*-glycans were profiled by ultra-performance liquid chromatography. Clinical data was collected to link glycomic findings with metabolic and hormonal profiles. Total serum glycoprotein and IgG glycosylation differed in patients with endometriosis compared to control cases. The most significantly altered was glycan peak 3 from IgG, containing bisected biantennary glycans, which was decreased in the endometriosis cohorts (p = 0.0000005–0.018). In conclusion, this is the first pilot study to identify changes in *N*-glycans from whole serum glycoproteins associated with endometriosis. A larger validation study is now warranted and such studies should include the follow-up of surgically and pharmacologically treated patients.

## Introduction

Endometriosis (EMS) is a chronic inflammatory gynaecological disease of unknown aetiology characterized by the growth of endometrial tissue outside the uterine cavity^1–5^. Affecting 6–10% of women of reproductive age, EMS is a leading cause of female infertility as well as causing significant dysmenorrhoea and pelvic pain^1,4^. It has also been associated with increased risks of cancer and autoimmune diseases^1,2,4^. The aetiology of EMS is multifactorial and implicated factors include genetic predisposition, prenatal exposure to endocrine-disrupting chemicals and alterations in the microbiome, the immune system and sex hormones^4,6^. Laparoscopy is the gold standard for definitive EMS diagnosis^1^. There are no comprehensive non-invasive diagnostic tools currently available^7^, although ovarian endometriomas and deep nodular forms of the disease can be detected by ultrasonography and MRI^3^. Treatment depends on surgical intervention and hormone therapy and recurrence rates are high^1^. Early recognition and treatment of EMS improves patient fertility, dysmenorrhoea and pelvic pain, thereby improving quality of life^1^. Furthermore, EMS represents a significant public health problem due to its effect on the quality of life of women^5^. This in turn causes a substantial economic burden with EMS-related costs including hospitalisation for the disease and lost days at work due to both symptoms and time needed for medical and surgical interventions^1^.

Hormones and metabolic features are dysregulated in EMS throughout the menstrual cycle^8,9^. In endometrioma tissue from women with EMS, there is significantly increased glucose consumption, lactate production and aberrant expression of glycolysis-related enzymes compared with healthy controls, suggesting that endometrioma is associated with enhanced cellular glycolytic metabolism, resembling the Warburg effect observed in cancer cells^10^. Expression of glucose transporters, namely the GLUT4 transporter, is also altered in eutopic and ectopic endometrial tissue and between women with and without EMS^11^.

Glycans decorate all eukaryotic cell surfaces and altered glycosylation patterns have been found in many diseases, including chronic inflammatory conditions and cancer^12^. As such, glycan profiles characteristic of disease have promising potential as both clinical markers and therapeutic targets in EMS^1^. Little has been published on EMS and glycosylation, and the majority of the studies used invasively collected tissue or peritoneal fluid samples^1^. Reported glycosylation changes in EMS include altered glycosylation of plasma glycoproteins in secretory phase endometrial tissue from women with advanced EMS compared to controls, aberrant glycosylation and expression of EMS-associated peritoneal haptoglobin and alterations in sialylation on endometrial cells and in sera of patients with EMS^1,13–15^. Serum sialylation levels were found to change with Zoladex (Goserelin Acetate) therapy, a treatment for EMS, suggesting that serum glycosylation could be a promising biomarker candidate of EMS^16^. A recent study found sialylation and galactosylation on IgG to be altered in EMS^13^. Changes in serum/plasma *N*-glycosylation occur mainly in acute phase proteins and IgG^12^. The aetiology of EMS is most likely multifactorial but immune factors are undoubtedly at play^17,18^. Based on our current knowledge, we believe that the development and persistence of the disease depends on several coexisting factors. However, focusing on immunoglobulins such as IgG may help to better understand this poorly understood chronic disease. In addition, IgG glycosylation is well studied and understood, making it easier to put observed differences into context than is the case with other high abundance proteins^19^. Additionally, at the whole serum level, small changes in these glycans may be masked by other glycans in the whole serum.

The aims of this study were to profile changes in *N*-glycans on whole serum glycoproteins and IgG, to relate them to the expression of hormones and glucose metabolism and to assess the potential of glycans for the non-invasive diagnosis of EMS. We used two European cohorts of patients with EMS and comparator non-EMS controls.

## Results

### Glycosylation on whole serum and serum IgG glycoproteins in EMS

**Figure 1:**
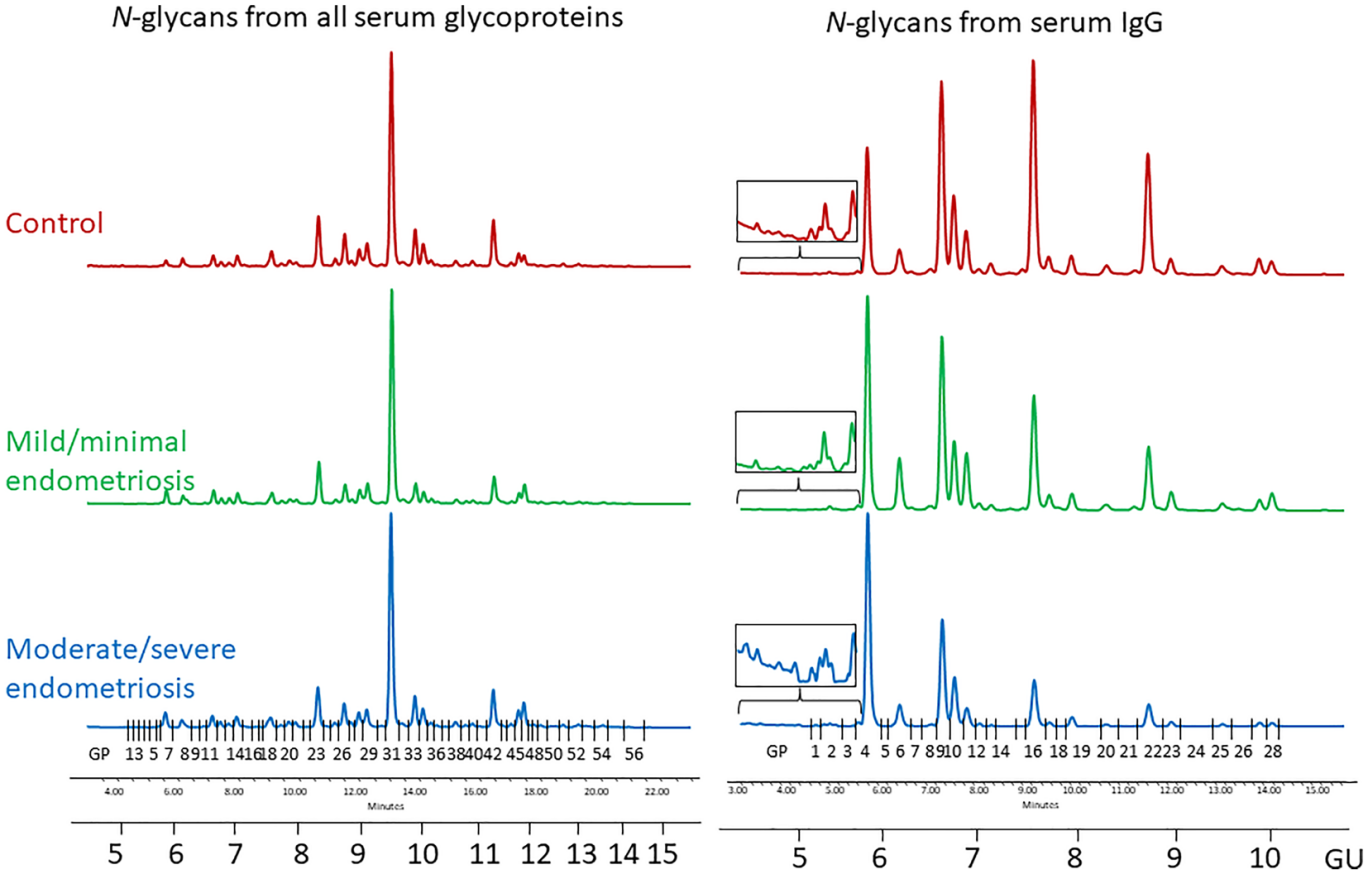
Representative HILIC-UPLC chromatograms of the *N*-glycans from all whole serum and IgG glycoproteins of control (without endometriosis, top) and endometriosis (minimal/mild and moderate/severe) samples. Each glycan peak (GP) was numbered and detailed information about the assigned structures are presented in Supplementary Tables S2 and S3. Boxes represent zoomed areas, on the x-axis are plotted glucose units (GUs) and on the y-axis is fluorescence.

The *N*-glycans from whole serum glycoproteins and from IgG were released from all samples (Supplementary Table S1) and profiled by Hydrophilic Interaction Liquid Chromatography—Ultra-Performance Liquid Chromatography (HILIC-UPLC). The resulting chromatograms were separated into 56 and 28 glycan peaks (GPs), respectively (Fig. 1, Supplementary Fig. S1). The glycans in each peak from serum and IgG *N*-glycans were assigned based on previous publications^20–22^. The glycans present in each GP and the associated glycan features are listed in Supplementary Tables S2 and S3.

Cohort 1 was more heterogeneous than cohort 2. More specifically, in several controls from this cohort laparoscopy was not done (7 women from this cohort had glycosylation data) and samples were collected both in the follicular and luteal phases of the menstrual cycle, whereas all subjects in cohort 2 were in the mid-luteal phase and were confirmed at laparoscopy to be EMS-free. Therefore, we divided all samples from cohort 1 into 3 subgroups (a) including only those who had a laparoscopy, (b) only those samples in the mid-luteal phase (c) patients who had a laparoscopy and were mid-luteal. These groups were analysed together as well as separately for *N*-glycan changes on serum and IgG proteins (Supplementary Tables S4 and S5). To further investigate any potential confounding impact of secondary inflammatory gynaecological conditions on glycosylation status, we also analysed subgroups after excluding subjects with the following: hydrosalpinx, salpingitis, sactosalpinx or inflammatory cysts on fallopian tubes (10 women from cohort 1 and 3 women from cohort 2).

**Figure 2:**
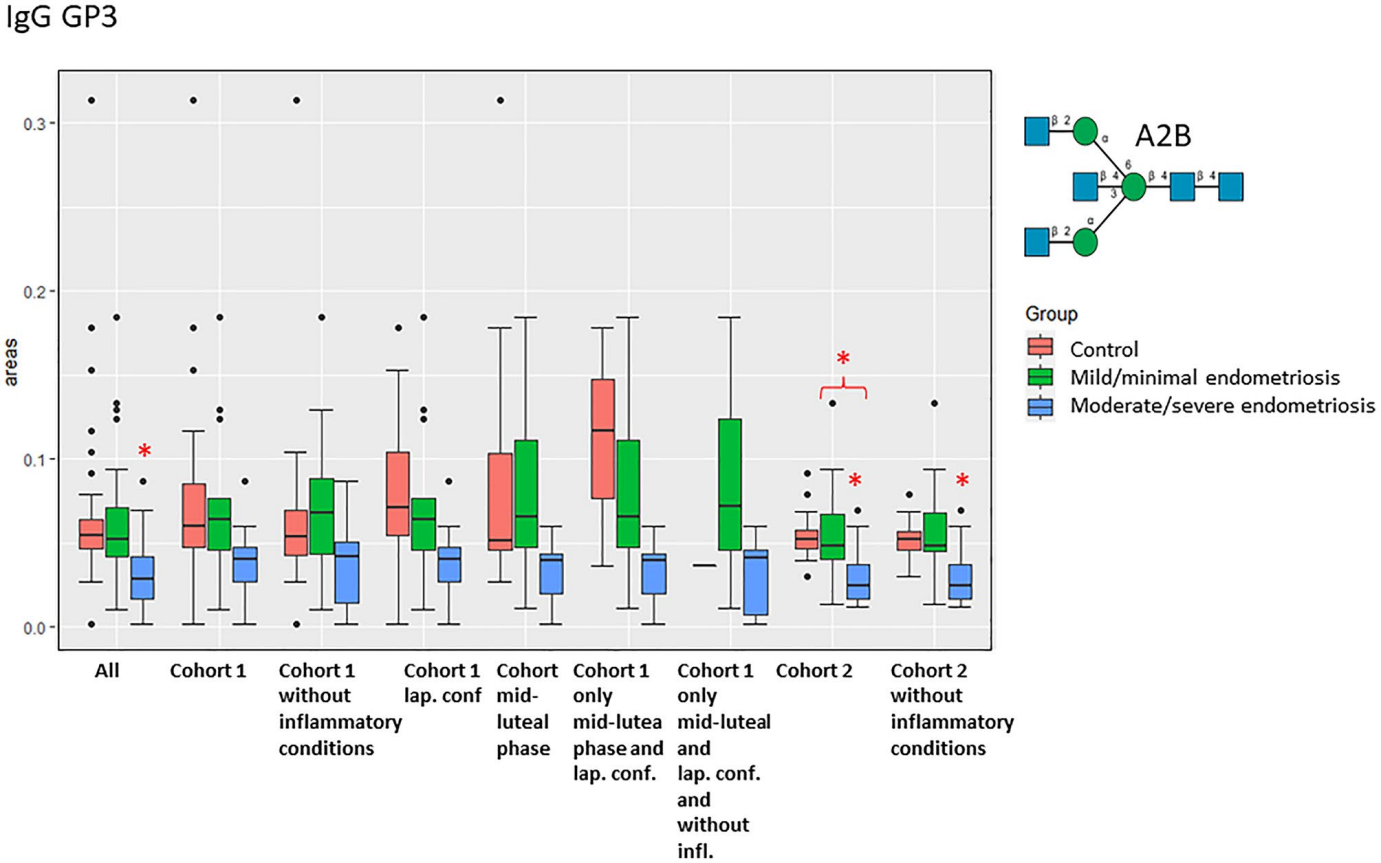
Boxplots for GP3 from IgG for each group. Significant comparisons after Bonferroni correction are starred (endometriosis patient groups in comparison with controls are starred). Boxes represent the 25th and the 75th percentiles with the median indicated. Statistically significant comparisons of the particular disease group with control group after Bonferroni correction for multiple testing are labelled with red star. More heterogenous cohort 1 is sub-separated into more subgroups.

We found significantly different amounts of particular GPs and features in EMS compared to controls, especially in the groups where other inflammatory gynaecological conditions had been excluded (Fig. 2, Supplementary Fig. S2, Supplementary Tables S4 and S5).

Specifically, in the *whole serum N-glycome* GP24 (biantennary bisected digalactosylated monosialylated glycans) was significantly increased in all EMS samples (p = 1.58 × 10^–6^) (Supplementary Fig. S2, Supplementary Table S4). When the EMS group was separated into minimal/mild and moderate/severe subgroups, this GP was significantly increased in the moderate/severe group in all samples (p = 3.02 × 10^–8^) and in cohort 2 (p = 5.10 × 10^–9^) but also in the minimal/mild group in cohort 2 (5.10 × 10^–9^) (Supplementary Fig. S2, Supplementary Table S4). GP22 (core fucosylated bisected monogalactosylated monosialylated glycans) was significantly increased in moderate/severe EMS in all samples (p = 0.0009) as well as in cohort 2 (p = 1.97 × 10^–7^) (Supplementary Fig. S2, Supplementary Table S4).

Many other GPs were altered only in cohort 2 group, with cohort 1 following the trend, mainly in the group consisting of only mid-luteal phase subjects (Supplementary Fig. S2). There were increases in GP15 (monoantennary monogalactosylated monosialylated and biantennary digalactosylated glycans) in moderate/severe EMS (p = 0.00005), S1 (monosialylated glycans) in the minimal/mild EMS without inflammatory conditions (p = 0.0014), and decreases in GP53 (mainly tetraantennary tetragalactosylated and tetrasialylated glycans) (p = 0.00087) in minimal/mild EMS, S3 (trisialylated glycans) in minimal/mild EMS (p = 0.0002), S4 (tetrasialylated glycans) in minimal/mild EMS (p = 0.0001) and outer arm fucosylated glycans in minimal/mild EMS without inflammatory conditions (p = 0.0004) compared to controls (Supplementary Table S4).

In the *serum IgG N-glycome*, GP3 was the most consistent between the cohorts and significant from all GPs (Fig. 2, Supplementary Table S5). It contains biantennary bisected glycan A2B (Supplementary Table S3). It gradually decreased with the EMS progression from minimal/mild to moderate/severe stage, being significantly decreased in moderate/severe EMS compared to controls (p = 0.000015) in all samples and in cohort 2 (p = 5.12 × 10^–7^) (Fig. 2, Supplementary Table S5). GP8 (mainly biantennary bisected monogalactosylated glycans) was also decreased in moderate/severe EMS (p = 0.00032) in all samples and in cohort 2 in moderate/severe EMS (p = 0.000084) (Supplementary Fig. S1, Supplementary Table S5). GP15 (core fucosylated biantennary digalactosylated glycans) was decreased in moderate/severe EMS in all samples (p = 4.04 × 10^–6^), and in moderate/severe EMS (p = 0.00029) compared to controls (Supplementary Fig. S2, Supplementary Table S5).

Overall, the changes in glycosylation sometimes reached significance only in one cohort, cohort 2, and not surprisingly, most peaks got more consistent results when only subjects in the mid-luteal phase from cohort 1 were selected. More significant results were found in cohort 2 possibly due to the higher numbers of subjects and lower heterogeneity. Importantly, IgG GP3 was significantly consistently decreased in all the EMS groups regardless of the cohort heterogeneity or the phase of the cycle (Fig. 2).

**Figure 3:**
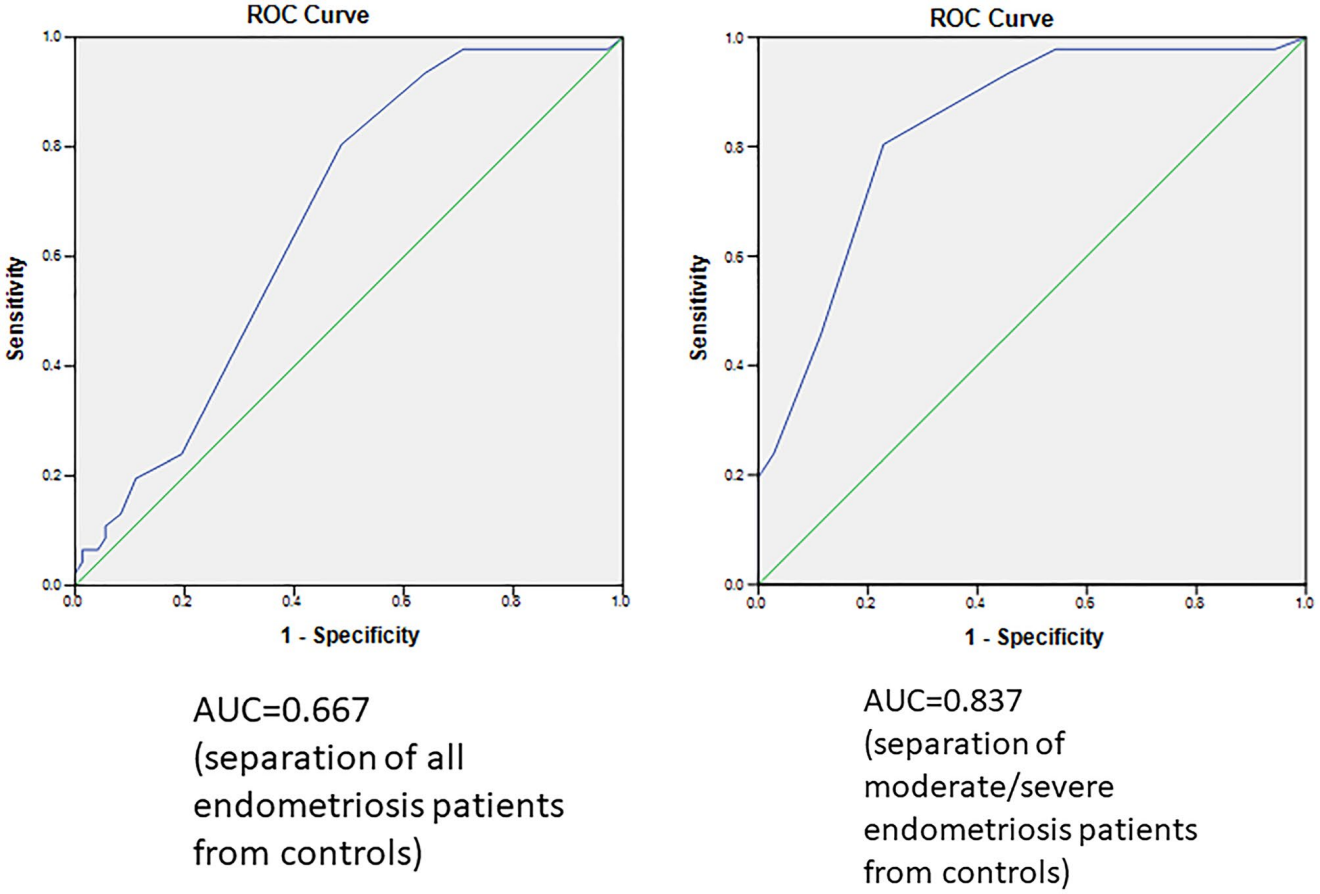
ROC curves for GP3 in whole patient cohort. More details including the points on the ROC curves and calculated cut-points are in the Supplementary Table S6.

IgG GP3 has the most promising biomarker potential for EMS. We have plotted the ROC curve on the whole cohort and the AUC was 0.667 when we separated all EMS from controls and 0.837 when separating moderate/severe EMS cases from controls (Fig. 3). Optimum cut-point values were calculated according to the IU method, which was selected as the best suited to balance sensitivity (true positive rate) and 1-specificity (false positive rate)^23^ (Supplementary Table S6). The optimum cut-point values for IgG GP3 value from the whole cohort to separate from controls was calculated to be 0.055 with corresponding values of 0.457 sensitivity and 0.694 specificity. The optimum cut-point for IgG GP3 value from the moderate/severe EMS cases to be separated from controls was calculated to be 0.045 with 0.804 sensitivity and 0.771 specificity.

### Correlation of glycans with hormones and glucose metabolism markers

GPs and features were correlated with the clinical factors, hormones and glucose metabolic markers. Although some significant correlations were found, the correlation coefficients were rather small (Supplementary Table S7).

The following correlations are of interest as they demonstrated correlations of significantly altered peaks in EMS.

*Cycle length correlated positively with* GP22 (core fucosylated bisected monogalactosylated monosialylated glycans) from serum *N*-glycans only in the luteal phase.

*Luteinizing hormone (LH) correlated negatively with* GP44 (core fucosylated trigalactosylated trisialylated glycans), GP50 (tetraantennary tetragalactosylated tetrasialylated glycans), GP52 (tetraantennary tetragalactosylated tetrasialylated glycans), A3 (triantennary glycans), G3 (trigalactosylated glycans), S3 (trisialylated glycans, only in the luteal phase) and S4 (tetrasialylated glycans) and *positively with* A2 (biantennary glycans) from serum *N*-glycans.

*Insulin correlated negatively with* GP15 (monoantennary monogalactosylated monosialylated and biantennary digalactosylated glycans) and S1 (monosialylated glycans) and *positively correlated with* outer arm fucosylated glycans from serum *N*-glycans.

*Age correlated negatively with* A2 (biantennary glycans) and *positively with* GP22 (core fucosylated bisected monogalactosylated monosialylated glycans), GP24 (biantennary bisected digalactosylated monosialylated glycans), GP50 (tetraantennary tetragalactosylated tetrasialylated glycans, only in the luteal phase), G3 (trigalactosylated glycans) and A3 (triantennary glycans) from serum *N*-glycans.

*Body mass index (BMI) correlated negatively with* GP11 (mainly core fucosylated biantennary monogalactosylated glycans), GP15 (monoantennary monogalactosylated monosialylated and biantennary digalactosylated glycans), S0 (non sialylated glycans), S1 (monosialylated glycans), G1 (monogalactosylated glycans), A2 (biantennary glycans) and *positively with* GP44 (core fucosylated trigalactosylated trisialylated glycans), S3 (trisialylated glycans), G3 (trigalactosylated glycans), A3 (triantennary glycans), outer arm fucosylated glycans from serum *N*-glycans was increased.

The phase of the menstrual cycle and the presence of other inflammatory factors didn’t significantly affect the important peaks when correction for multiple testing was taken into account. Follicle stimulating hormone (FSH), oestradiol, progesterone, fasting glucose and haemoglobin A1c (HbA1c) levels did not correlate with any significant glycans.

### Hormones and metabolic markers in EMS

Patient characteristics are summarised in Table 1A. Median age and BMI did not significantly differ amongst groups. Hormones (LH, FSH, oestradiol) and glucose metabolic markers (insulin, fasting glucose and HbA1c) also did not differ among groups. There was a lower progesterone concentration in patients with EMS in the luteal phase, but this was not deemed to be statistically significant (p > 0.05). This might be due to more frequent anovulatory cycles with lower progesterone concentrations in EMS (Supplementary Table S1, Table 1B). Cycles were classified as anovulatory when the progesterone concentration was below 5.3 nmol/L, despite being taken at what was thought to be the mid-luteal phase of the cycle. Samples deemed anovulatory for 2 women in the control group of cohort 1 (10%, 5.6% from pooled cohorts), 4 women with minimal/mild EMS from cohort 1 (36.4%, 20% from pooled cohorts) and 2 women from cohort 1 and 1 women from cohort 2 with moderate/severe EMS (25%, 6.7% and 13% from pooled cohorts).

**Table 1:** Clinical characteristics summary.

(A) In the whole cohort						
Clinical characteristics	Controls		Minimal/mild endometriosis		Moderate/severe endometriosis	
Phase of the cycle (sample numbers)	Follicular (8, cohort 1)	Luteal (45, 20 cohort 1 and 25 cohort 2)	Follicular (3, cohort 1)	Luteal (34, 12 cohort 1 and 22 cohort 2	Follicular (5, cohort 1)	Luteal (31, 8 cohort 1 and 23 cohort 2)
Age (years)	38, 35–38	32, 29–36	37, 29–39	33, 30–34	32, 30–40	34, 30–37
BMI (kg/m^2^)	22.5, 20.2–22.9	22.0,20.7–25.0	23.3, 22.8–23.5	22.2, 20.0–23.5	25.6, 20.7–25.9	22.0, 20.6–24.8
Cycle length	28, 27–28	28, 27–31	31, 29–32	27, 26–28	30, 28–30	29, 29–29
LH (IU/L)	9.4, 8.4–12.8	5.8, 3.6–8.5	5.9, 5.6–6.2	7.1, 3.6–15.0	9.3, 3.5–10.9	5.7, 4.9–8.7
FSH (U/L)	7.1, 6.2–10.9	3.2, 2.7–4.5	8.9, 7.8–9.9	3.9, 3.6–5.5	6.2, 3.5–6.9	3.3, 2.2–4.5
Oestradiol (pmol/L)	2.97.4, 145.0–464.9	562.5, 516.7–729.9	413.5*	484.6, 300.0–671.4	263.5, 174.3–396.2	475.3, 316–753.4
Progesterone (nmol/L)	1.9, 0.5–3.4	39.7, 34.1–57.3	0.8, 0.7–1.0	28.1, 12.7–51.5	2.7, 0.5–5.0	32.3, 16.9–46.8
Insulin (mU/L)	5.4, 4.6–6.0	7.7, 5.5–10.5	3.2, 1.9–4.5	6.4, 4.0–7.9	6.0, 4.4–8.9	6.5, 4.4–13.5
Fasting glucose (mmol/L)	4.7, 4.5–5.1	4.5, 4.3–5.0	4.3, 4.2–4.5	5.4, 4.6–6.1	5.8, 5.2–6.2	4.9, 4.5–5.4
HbA1c (mmol/mol)	31.5, 30.3–35.8	35.0, 32.0–35.0	30.0, 29.5–30.5	35.0, 33.0–35.0	32.0, 30.0–32.0	36.0*

(B) In the luteal phase patients, focused on the oestradiol to progesterone levels and ovulation						
Clinical characteristics	Controls		Minimal/mild endometriosis		Moderate/severe endometriosis	
Ovulation (sample numbers)	All (10)	Ovulatory (9)	All (10)	Ovulatory (6)	All (6)	Ovulatory (4)
Oestradiol/progesterone ratio	13.0,10.3–21.4	13.0, 9.8–15.3	25.1, 18.7–342.1	19.0, 13.6–23.3	29.0, 17.8–86.0	19.7, 15.6–26.3

Data are presented as medians, and interquartile ranges (all clinical details are in Supplementary Table S1).

Age and BMI were compared on the whole cohort, the hormones were compared only among cases in mid-luteal cycle.

*Only one case in this group.

The criterion for significance was set at p value ≤ 0.05 (no significant differences among any groups were found).

If we look at the oestradiol to progesterone ratio only in the ovulatory cases, we see an increase in the EMS groups, although this is not statistically significant, specifically 13.0 in controls, 19.0 in minimal/mild EMS and 19.7 in moderate/severe EMS (Table 1B). However, if the anovulatory cases are included, the pooled EMS group has a significantly higher ratio of oestradiol to progesterone (p = 0.023), namely, 13.0 in controls, 25.1 in minimal/mild EMS and 29.0 in moderate/severe EMS (Table 1B).

### Separation of women with inflammatory gynaecological conditions based on all *N*-glycans

**Figure 4:**
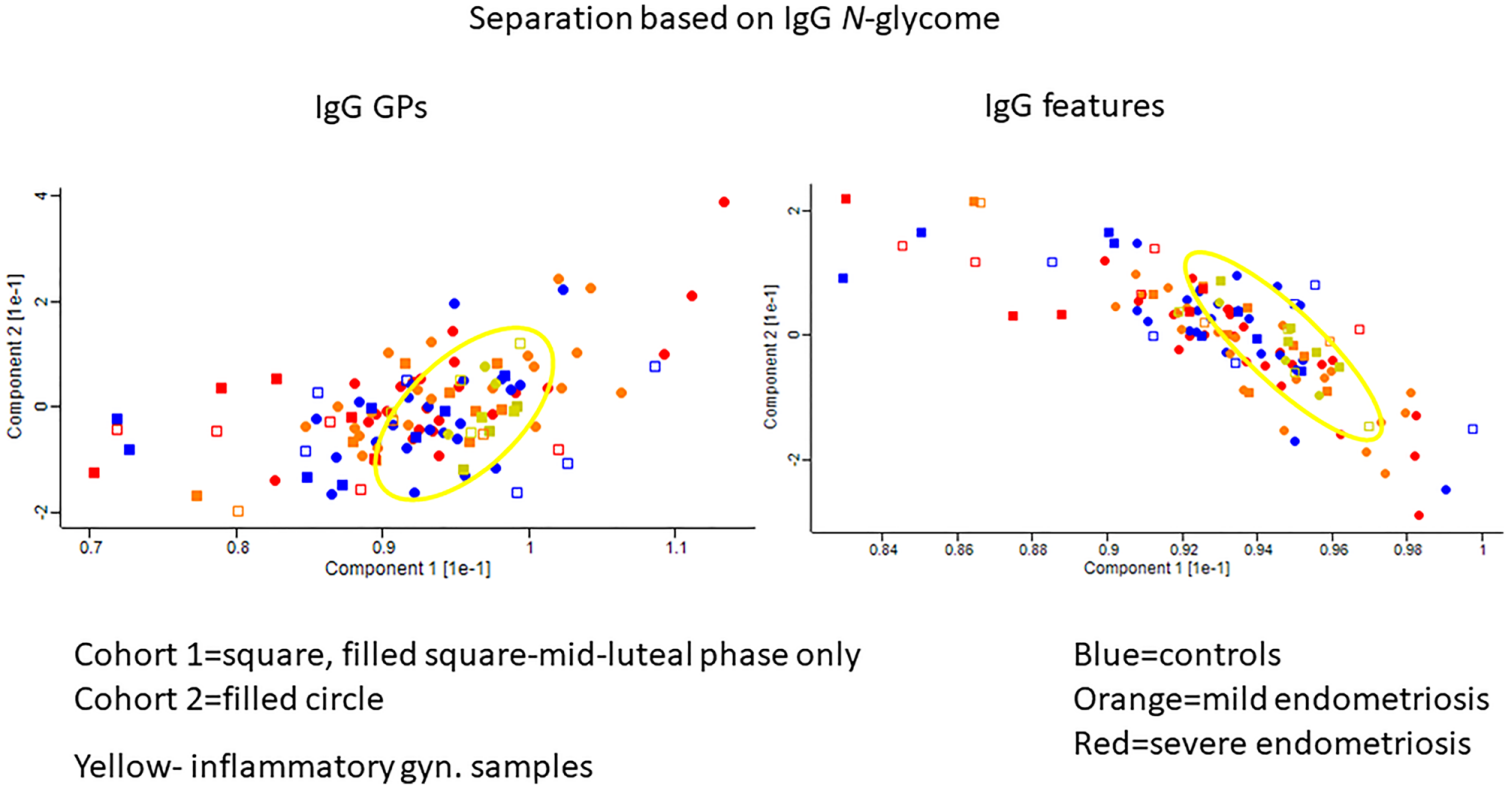
Clustering of all subjects based on IgG *N*-glycome. In the figure: Blue = controls, Orange = minimal/mild endometriosis, Red = moderate/severe endometriosis, Yellow= inflammatory gynaecological samples.

Using PCA plots to separate EMS patients from controls using all GPs generated from whole serum and serum IgG *N*-glycan profiles, the controls and EMS cases were not well separated (Fig. 4, Supplementary Fig. S3). However, in case of the IgG, we found that the subjects with gynaecological inflammatory conditions cluster together although they are not completely separated from the other subjects (Fig. 4, Supplementary Fig. S4).

## Discussion

The diagnosis of EMS remains challenging, due to the wide spectrum of symptoms associated with the disease and the frequent overlap of these symptoms with other gynaecological and medical conditions^1^. Definitive diagnosis is performed by invasive laparoscopic surgery^1^ although ovarian endometriomas and deep nodular forms of the disease can be detected via ultrasonography and MRI^3^. Numerous attempts have been made to develop an effective and less invasive diagnostic method to date; however, there are currently no non-invasive biomarker candidates which can replace that invasive laparoscopy^1,7^. Serum is obtained relatively non-invasively and could provide and excellent source of non-invasive and long-awaited biomarkers.

This is the first study on glycosylation from whole serum glycoproteins in patients with EMS. We have compared women with EMS to controls who present with symptoms or infertility, which could potentially lead to valuable clinical diagnostic markers as the challenge is to separate EMS patients from the other patients with similar complaints rather than from healthy controls.

We found significant differences in particular *N*-glycans from whole serum glycoproteins and IgG in patients with EMS compared to controls without the disease. The group of glycans most differentially expressed in the EMS group was GP3 from IgG, containing bisecting biantennary glycans. The AUC was better for severe EMS patients (0.837) comparing to the entire pooled EMS group (0.667), showing diagnostic potential. The optimum cut-point values for IgG GP3 values from the whole EMS group to separate them from controls was calculated to be 0.055 and to separate moderate/severe EMS patients from controls was calculated to be 0.045 with ‘optimal’ sensitivities and specificities. The cut-point values falling towards the left on the curve from these cut-points have lower false positive rates but also lower true positive rates, and, on the right side, higher true positive rates but also higher false positive rates.

Additionally, this peak was significantly consistently decreased in the EMS groups regardless of cohort heterogeneity or phase of the cycle. Bisecting glycans decreased with age^12^. The decrease in bisecting glycans in EMS may be associated with hormone imbalance, which is more pronounced in the later stages of EMS. Using all glycans and/or the derived features generated from whole serum or serum IgG *N*-glycomes didn’t separate EMS from controls.

Interestingly, highly sialylated, galactosylated and outer arm fucosylated serum glycans are decreased in EMS, which is in contradiction to common changes with inflammatory conditions^12^. While EMS is widely considered an inflammatory condition^1,3,5^, dysregulated levels of oestradiol and progesterone associated with the disease may influence these particular glycosylation changes.

Reduced sialylation and galactosylation and an increase in agalactosylated glycans was observed on IgG from patients with EMS compared to healthy women, but not compared to women with other gynaecological conditions^13^. In this study only an increase in agalactosylated glycans on IgG in one of the groups with EMS was found, but it did not reach significance after correction for multiple testing.

Inflammatory conditions influence protein glycosylation^12^, and this is a likely explanation for the clustering of the inflammatory cases based on the IgG glycome. As EMS is a chronic inflammatory condition^1,3,5^, it is not surprising that it has an effect on the IgG glycosylation.

Quite often, the data is more consistent when only mid-luteal samples from cohort 1 are selected showing a likely effect of hormones on glycosylation. However, further validation in a larger study is needed.

We found weak correlations between some glycan peaks and clinical characteristics, hormones and glucose metabolism markers. Most correlations were found with age and BMI, some correlations were found with cycle length, FSH, LH and insulin levels, but the control and EMS groups did not differ significantly in these factors. It is therefore unlikely that these correlations would contribute to the significant changes found in EMS groups compared to controls. Fasting glucose, HbA1c, FSH, oestradiol and progesterone concentrations did not correlate with any significant glycans. Phase of the cycle and inflammation did not significantly affect the important peaks when correction for multiple testing was taken in account*.*

We found a decreased trend in serum progesterone levels in patients with EMS. There were no significant changes among groups in hormones and glucose metabolism markers. This is in agreement with another study which found no significant differences in serum levels of oestradiol and progesterone in EMS^24^. Oestradiol has been shown to increase with EMS progression^25^, but levels did not differ in EMS compared to controls^26^. Interestingly, when we have looked at the oestradiol to progesterone ratio, there was a significant increase in the EMS group. However, when we excluded anovulatory women, more represented in the EMS groups^27^, this difference was no longer evident. This suggests over-exposure to oestradiol and under-exposure to progesterone in EMS. This may also explain why progesterone is effective in EMS treatment. However, progesterone resistance is implicated in EMS^28,29^.

## Conclusions

Strengths of this study include the fact that we utilised two independent cohorts from two separate institutions and that we compared EMS cases with other gynaecological patients with similar symptoms, rather than healthy controls. While serum glycome testing in EMS patients and healthy/fertile women might show greater EMS glycosylation differences, we believe it is more clinically valuable to separate EMS from other patients with similar complaints. Our controls represented patients undergoing laparoscopy, because of a suspected diagnosis of EMS, but proven to be EMS free. These patients may suffer from several underlying conditions related to pelvic inflammatory disease and this was allowed for by our study design.

As this was an exploratory pilot study, it is limited by the low subject numbers. The slight differences in the sample collection protocols between the two cohorts is also a minor limitation.

We conclude that EMS-associated IgG-GP3 may represent a viable target for the development of innovative non-invasive or minimally invasive diagnostic tools for the diagnosis of EMS. A serum test, possibly in the lectin-ELISA setting, and/or as part of a panel of other promising protein or other biomarkers^1^ would be easily transferable to clinical settings. Following on from this pilot study, a larger study with greater numbers and tighter control of clinical factors such as menstrual cycle stage is indicated in the quest for a potential future biomarker for EMS.

## Materials and methods

### Ethics approval, guidelines, and consent to participate

The study protocol was approved by the Research Ethics Committee of the National Maternity Hospital, Dublin (Ref. No.:EC19.2018; *Cohort 1*) and the Research Ethics Committee of the University of Tartu (337/T-5, 15.03.2021; *Cohort 2*). All procedures were performed in accordance with the Declaration of Helsinki. Written informed consent was obtained from all participants.

### Patients

Summary data of all women and their clinical characteristics are shown in Table 1 and Supplementary Table S1. Two cohorts of patients and controls from two individual institutions were studied. All EMS patients and 17 controls from cohort 1 and all patients and controls from cohort 2 underwent laparoscopy for the investigation of infertility, pelvic pain or pelvic pathology suspected on ultrasound scan. All subjects had an ultrasound performed by an operator skilled in gynaecological imaging. Post laparoscopy, all women were classified as no EMS (controls, 17 from cohort 1 and 25 from cohort 2), or EMS and the severity of EMS was classified according to the revised American Society for Reproductive Medicine classification system^7^ into minimal/mild EMS (ASRM stage 1/2, n = 38, 16 from cohort 1 and 22 from cohort 2) and moderate/severe EMS (ASRM stage 3/4, n = 37, 14 from cohort 1 and 23 from cohort 2). Cohort 1 also contained another 17 controls who did not undergo laparoscopy (due to a lack of EMS symptoms or any ultrasound findings suggestive of EMS). None of the women had been using any hormonal medications (GnRH analogues, progestogens, combined oral contraceptives) and/or anti-inflammatory therapy for at least three months. All women had negative screening results for sexually transmitted diseases, active systemic infection, a history of autoimmune diseases, active vaginosis; acquired or primary immunodeficiency diseases (including HIV), pregnancy, or a malignant condition. Inflammatory gynaecological conditions diagnosed by laparoscopy, such as hydrosalpinx, salpingitis, sactosalpinx, polycystic ovarian syndrome (PCOS) and inflammatory cysts, were noted, as well as use of antibiotics and phase of the menstrual cycle.

The phase of the menstrual cycle was assigned as follicular or luteal based on the patient’s average cycle length and the day of the cycle on which laparoscopy (and sample collection) was performed. In the 17 controls from cohort 1 who did not have laparoscopy, samples were taken at the had timed mid-secretory phase, timed 6–8 days after a urinary luteinizing hormone peak.

### Sample collection

Peripheral blood samples were collected using BD Vacutainer® Venous Blood Collection tubes containing clotting activator (BD Company, Franklin Lakes, New Jersey, U.S.) before anaesthesia. Samples were processed within 1 h after collection. Serum was isolated from the peripheral whole blood samples by centrifugation at 2000×*g*/10 min/4 °C (cohort 1) or two centrifugations at 1600×*g* for 10 min and at 16,000×*g* for 10 min at 4 °C (cohort 2). Separated serum samples were stored at − 80 °C until use. Endometrial biopsies were collected at laparoscopy or in the mid-luteal phase for those not undergoing laparoscopy.

### Hormone and glucose metabolism markers measurements

Luteinizing hormone (LH), follicle stimulating hormone (FSH), progesterone, insulin and haemoglobin A1c (HbA1c) were measured using routine clinical lab tests. Whole blood was collected into lavender top vacutainers with ethylenediaminetetraacetic acid (EDTA) anticoagulant for HbA1c analysis. For fasting glucose, blood was collected into grey top tubes (with sodium fluoride, glycolytic inhibitor). Serum samples were used to measure LH, FSH, oestradiol, progesterone and insulin.

### Isolation of IgG

50 µL serum was used to isolate IgG. The serum IgG was captured using Protein G PhyTips (PTH 91-20-02 Box of 96 PhyTip columns) and a Buffer kit of PhyNexus (BUF-91-40-01) (Biotage, Uppsala, Sweden). Pre-equilibrated PhyTips were used for IgG capture (equilibration with Buffer A solution 200 µL per well, 20 cycles, at lowest speed; IgG capture with 200 µL Buffer A solution and 50 µL serum sample, 30 cycles, at lowest speed). The captured IgG in the PhyTip resin was washed with Wash buffer I (250 µL per well mixing volume, 10 cycles, at lowest speed) followed by a treatment of Wash buffer II (250 µL per well mixing volume, 10 cycles, at lowest speed). Then, IgG was eluted (250 µL per well mixing volume, 20 cycles, at lowest speed). Samples were neutralized with TRIS buffer, pH 11.0 (MERCK, NJ, USA). All solutions used for the process were from the Buffer kit of PhyNexus except the 0.1 M citric acid reagent, pH 2.5 used for elution (MERCK, NJ, USA), as this solution provided the best result after Peptide-*N-*Glycosidase F (PNGase F) treatment.

### *N*-glycan release from whole serum glycoproteins and from isolated IgG

Glycans were released from 5 µL of serum samples and from the isolated IgG and dissolved in 10 µL of double distilled water (ddH_2_O) before using the high-throughput method described by Royle et al.^30,31^. Briefly, the samples were reduced and alkylated in 96-well plates, immobilized in SDS-gel blocks, and then washed. The *N*-glycans were released using peptide *N*-glycanase F (1000 U/mL; NEB, cat. number P0709L), as previously described^32^. Neutralized IgG samples were centrifuged through a 10 kDa filter (Pall Corporation, NY, USA) at 10 min, 14,000×*g* then 100 µL of denaturation buffer [(50 mM dithiothreitol (DTT), 20 mM NaHCO_3_, 0.1% SDS) (MERCK, NJ, USA)] was added to the filter and incubated at 20 min, 65 °C. After denaturation 40 µL of 0.1 M iodoacetamide was added to each sample and incubated at 15 min, 40 °C (MERCK, NJ, USA). At the end of the incubation, samples were centrifuged at 10 min, 14,000×*g*, then 100 µL ddH_2_O was added to the samples at 10 min, 14,000×*g*. 20 µL PNGase F mix was added to each sample and incubated at 20 min, 37 °C to release *N*-glycans [20 mM NaHCO_3_, PNGase F concentration: 0.5 µL Prozyme (1000 U/mL)] in 20 µL buffer. After PNGase F treatment, the released glycans were centrifuged at 10 min, 14,000×*g* and collected into fresh Eppendorf tubes and dried in a SpeedVac system (Thermo Fisher Scientific, MA, USA).

### 2-Aminobenzamide (2-AB) labelling of glycans

The *N*-glycans were fluorescently labelled with 2-AB (MERCK, NJ, USA) by reductive amination^33^. This classical glycan labelling method involves a two-step process. The first step is the Schiff's base formation, where the primary amino group of the dye performs a nucleophilic attack on the carbonyl carbon of the acyclic reducing terminal residue to form a partially stable Schiff's base. Then, the Schiff's base imine group is chemically reduced with sodium cyanoborohydride to form a stable labelled glycan^34^. 2-AB labels glycans in a 1:1 stoichiometry in a structurally unbiased manner, which allows accurate quantitative measurements and comparison between samples^31,35^. Five µL of 2-AB was added to the released and dried glycans and labelled at 65 °C, 2 h in dark. Excess 2-AB was removed on Whatman 3MM paper (Clifton, NJ, USA) in acetonitrile^30,31^. While more sensitive labelling techniques are now available than the classical 2-AB labelling method, the major advantage of 2-AB is that an extensive glycan database is available only for the identification of 2-AB labelled structures^36^.

### Hydrophilic interaction liquid chromatography—ultra-performance liquid chromatography (HILIC-UPLC)

HILIC-UPLC was performed using a BEH Glycan 1.7 μm and 130 Å particles in 2.1 × 150 mm column (Waters, MA, US) on an Acquity UPLC (Waters, MA, US). Solvent A was 50 mM formic acid adjusted to pH 4.4 with ammonia solution and solvent B was acetonitrile. In the case of the whole serum samples, a 30 min method was used with a linear gradient of 30–47% with buffer A at 0.56 mL/min flow rate for 23 min followed by 47–70% buffer A and finally reverting back to 30% buffer A to complete the run method. In the case of the serum IgG samples, a 20 min method was applied with a linear gradient of 30–47% with buffer A at 0.56 mL/min flow rate for 16 min followed by 47–70% buffer A and finally reverting to 30% buffer A to complete the separation method. Samples were injected in 70% acetonitrile. 2-AB labelled fluorescence was detected at 420 nm with excitation at 330 nm. The system was calibrated using an external standard of hydrolyzed and 2-AB-labeled glucose oligomers to create a dextran ladder, as described previously^30^. The retention times of all identified peaks were given as glucose units (GU).

### Structure abbreviations

All *N*-glycans have two core *N*-acetylglucosamines (GlcNAcs); F at the start of the abbreviation indicates a core-fucose α1,6-linked to the inner GlcNAc; Mx, number (x) of mannose on core GlcNAcs; Ax, number of antenna (GlcNAc) on trimannosyl core; A2, biantennary with both GlcNAcs as β1,2-linked; A3, triantennary with a GlcNAc linked β1,2 to both mannose and the third GlcNAc linked β1,4 to the α1,3 linked mannose; A4, GlcNAcs linked as A3 with additional GlcNAc β1,6 linked to α1,6 mannose; B, bisecting GlcNAc linked β1,4 to β1,3 mannose; Gx, number (x) of β1,4 linked galactose on antenna; F(x), number (x) of fucose linked α1,3 to antenna GlcNAc; Sx, number (x) of sialic acids linked to galactose; Lac(x), number (x) of lactosamine (Galβ1-4GlcNAc) extensions.

### Glycan feature analysis

All glycans from serum and IgG proteins were pooled into groups based on their glycan composition, based on sialylation (S0 = non sialylated, S1 = monosialylated, S2 = disialylated, S3 = trisialylated and S4 = tetrasialylated glycans), galactosylation (G0 = agalactosylated, G1 = monogalactosylated, G2 = digalactosylated, G3 = trigalactosylated and G4 = tetragalactosylated glycans), branching (A1 = monoantennary, A2 = biantennary, A3 = triantennary and A4 = tetraantennary glycans) and fucosylation (core and outer arm fucosylated glycans), and bisecting (B) and oligomannosylated glycans were grouped (more details and explanation how these glycans were grouped is described and illustrated in Supplementary Tables S2 and S3).

### Statistical analyses

Statistical analyses were performed using SPSS statistical software for Windows (version 24.0; SPSS Inc.). Boxplots were generated in R studio (version 4.1.1). Glycan HILIC-UPLC data represent the relative percentage areas derived from the chromatographic profiles. The logit transform was used to map the data onto a more normal distribution: logit(peak) = log((peak/(1 − peak)). A multivariate analysis test (MANOVA) was used to assess differences in values (glycan peaks (GPs) and derived glycan features) between cases and controls followed by the use of the Tukey post hoc test. Nonparametric tests were used to evaluate if clinical data were significantly different among three groups (controls, patients with minimal/mild and moderate/severe EMS, Kruskal Wallis test) and two groups (controls and patients with EMS, Mann–Whitney test). Correlation among the clinical factors and GPs was done using Pearson correlation. The criterion for significance was set at *p value ≤ 0.05 or **p value ≤ 0.005 and ***p value ≤ 0.001. P-values were corrected for multiple testing by Bonferroni. Principal component analysis (PCA) was performed on % peak area and % feature area of each sample/group using the Perseus software platform (version 2.0.3.0) to identify relatedness of patient groups and samples.

### ROC curves

ROC curves, AUCs and ROC cut-point values were generated in SPSS statistical software for Windows (version 24.0; SPSS Inc.). The optimal cut-point values were calculated as minimum of IU (c) function according to IU method selected^23^: IU (c) = (|Se(c) – AUC| + |Sp(c) – AUC|), where Se is sensitivity and Sp specificity for the given point.

## Supplementary Information

Supplementary Figure S1.

Supplementary Figure S2.

Supplementary Figure S3.

Supplementary Figure S4.

Supplementary Tables.

## Data Availability

The data underlying this article are available in the article and in its supplementary material.

## References

[1] Kovács Z, Glover L, Reidy F, MacSharry J, Saldova R (2021). Novel diagnostic options for endometriosis: Based on the glycome andmicrobiome. J. Adv. Res..

[2] Mikhaleva LM (2021). Current knowledge on endometriosis etiology: A systematic review of literature. Int. J. Womens Health.

[3] Zondervan KT (2018). Endometriosis. Nat. Rev. Dis. Primers.

[4] Arafah M, Rashid S, Akhtar M (2021). Endometriosis: A comprehensive review. Adv. Anat. Pathol..

[5] Chapron C, Marcellin L, Borghese B, Santulli P (2019). Rethinking mechanisms, diagnosis and management of endometriosis. Nat. Rev. Endocrinol..

[6] Garcia-Penarrubia P, Ruiz-Alcaraz AJ, Martinez-Esparza M, Marin P, Machado-Linde F (2020). Hypothetical roadmap towards endometriosis: prenatal endocrine-disrupting chemical pollutant exposure, anogenital distance, gut-genital microbiota and subclinical infections. Hum. Reprod. Update.

[7] Kiesel L, Sourouni M (2019). Diagnosis of endometriosis in the 21st century. Climacteric.

[8] Hock DL, Sharafi K, Dagostino L, Kemmann E, Seifer DB (2001). Contribution of diminished ovarian reserve to hypofertility associated with endometriosis. J. Reprod. Med..

[9] Stilley JA, Birt JA, Sharpe-Timms KL (2012). Cellular and molecular basis for endometriosis-associated infertility. Cell Tissue Res..

[10] Qi X (2014). Knockdown of prohibitin expression promotes glucose metabolism in eutopic endometrial stromal cells from women with endometriosis. Reprod. Biomed. Online.

[11] McKinnon B (2014). Glucose transporter expression in eutopic endometrial tissue and ectopic endometriotic lesions. J. Mol. Endocrinol..

[12] Marino K, Saldova R, Adamczyk B, Rudd PM (2012). Carbohydrate Chemistry: Chemical and Biological Approaches.

[13] Solkiewicz K, Krotkiewski H, Jedryka M, Kratz EM (2021). Variability of serum IgG sialylation and galactosylation degree in women with advanced endometriosis. Sci. Rep..

[14] Solkiewicz K, Krotkiewski H, Jedryka M, Czekanski A, Kratz EM (2022). The alterations of serum IgG fucosylation as a potential additional new diagnostic marker in advanced endometriosis. J. Inflamm. Res..

[15] Solkiewicz K, Kacperczyk M, Krotkiewski H, Jedryka M, Kratz EM (2022). O-glycosylation changes in serum immunoglobulin G are associated with inflammation development in advanced endometriosis. Int. J. Mol. Sci..

[16] Rasha Z, Jasim BHA (2014). Sialic acid is a novel biochemical marker in sera of Iraqi endometriotic patients. J. Nat. Sci. Res..

[17] Ahn SH (2015). Pathophysiology and immune dysfunction in endometriosis. Biomed. Res. Int..

[18] Zhang T, De Carolis C, Man GCW, Wang CC (2018). The link between immunity, autoimmunity and endometriosis: A literature update. Autoimmun. Rev..

[19] Flevaris K, Kontoravdi C (2022). Immunoglobulin G N-glycan biomarkers for autoimmune diseases: Current state and a glycoinformatics perspective. Int. J. Mol. Sci..

[20] Saldova R (2014). Association of N-glycosylation with breast carcinoma and systemic features using high-resolution quantitative UPLC. J. Proteome Res..

[21] Stockmann H (2016). IgG N-glycosylation galactose incorporation ratios for the monitoring of classical galactosaemia. JIMD Rep..

[22] Pucic M (2011). High throughput isolation and glycosylation analysis of IgG-variability and heritability of the IgG glycome in three isolated human populations. Mol. Cell Proteomics.

[23] Unal I (2017). Defining an optimal cut-point value in ROC Analysis: An alternative approach. Comput. Math. Methods Med..

[24] Hapangama DK (2008). Endometriosis is associated with aberrant endometrial expression of telomerase and increased telomere length. Hum. Reprod..

[25] Ashish A, Kusum K, Rai S, Kumar B, Singh R (2021). Elevated levels of CA-125, estradiol and cortisol as prominent markers to diagnose various stages of endometriosis in Indian population. Int. J. Med. Res. Health Sci..

[26] Kianpour M, Nematbakhsh M, Ahmadi SM (2015). Asymmetric dimethylarginine (ADMA), nitric oxide metabolite, and estradiol levels in serum and peritoneal fluid in women with endometriosis. Iran J. Nurs. Midwif. Res..

[27] Soules MR, Makinak LR, Bury R, Poindexter A (1976). Endometriosis and anovulation: A coexisting problem in the infertile female. Am. J. Obstet. Gynecol..

[28] MacLean JA, Hayashi K (2022). Progesterone actions and resistance in gynecological disorders. Cells.

[29] Li Y (2016). Progesterone alleviates endometriosis via inhibition of uterine cell proliferation, inflammation and angiogenesis in an immunocompetent mouse model. PLoS ONE.

[30] Royle L, Radcliffe CM, Dwek RA, Rudd PM (2006). Detailed structural analysis of N-glycans released from glycoproteins in SDS-PAGE gel bands using HPLC combined with exoglycosidase array digestions. Methods Mol. Biol..

[31] Royle L (2008). HPLC-based analysis of serum N-glycans on a 96-well plate platform with dedicated database software. Anal. Biochem..

[32] Kuster B, Wheeler SF, Hunter AP, Dwek RA, Harvey DJ (1997). Sequencing of N-linked oligosaccharides directly from protein gels: In-gel deglycosylation followed by matrix-assisted laser desorption/ionization mass spectrometry and normal-phase high-performance liquid chromatography. Anal. Biochem..

[33] Bigge JC (1995). Nonselective and efficient fluorescent labeling of glycans using 2-amino benzamide and anthranilic acid. Anal. Biochem..

[34] Ruhaak LR (2010). Glycan labeling strategies and their use in identification and quantification. Anal. Bioanal. Chem..

[35] Marino K, Bones J, Kattla JJ, Rudd PM (2010). A systematic approach to protein glycosylation analysis: A path through the maze. Nat. Chem. Biol..

[36] Keser T, Pavic T, Lauc G, Gornik O (2018). Comparison of 2-aminobenzamide, procainamide and rapifluor-MS as derivatizing agents for high-throughput HILIC-UPLC-FLR-MS N-glycan analysis. Front. Chem..

