# Supplementary material for: N-glycans from serum IgG and total serum glycoproteins specific for endometriosis
Source: Sci Rep. 2023 Jun 28;13:10480. doi: 10.1038/s41598-023-37421-5 (PMC10307818; doi:10.1038/s41598-023-37421-5)
Supplement: Supplementary file 1 — Supplementary Figure S1. [file 41598_2023_37421_MOESM1_ESM.pdf]

Supplementary Figure S1. Representative HILIC-UPLC chromatograms of the *N*-glycans from whole serum glycoproteins and IgG of moderate/severe endometriosis sample with assigned major glycans in each glycan peak (GP). Detailed information about the assigned structures is presented in Supplementary Tables S2 and S3.

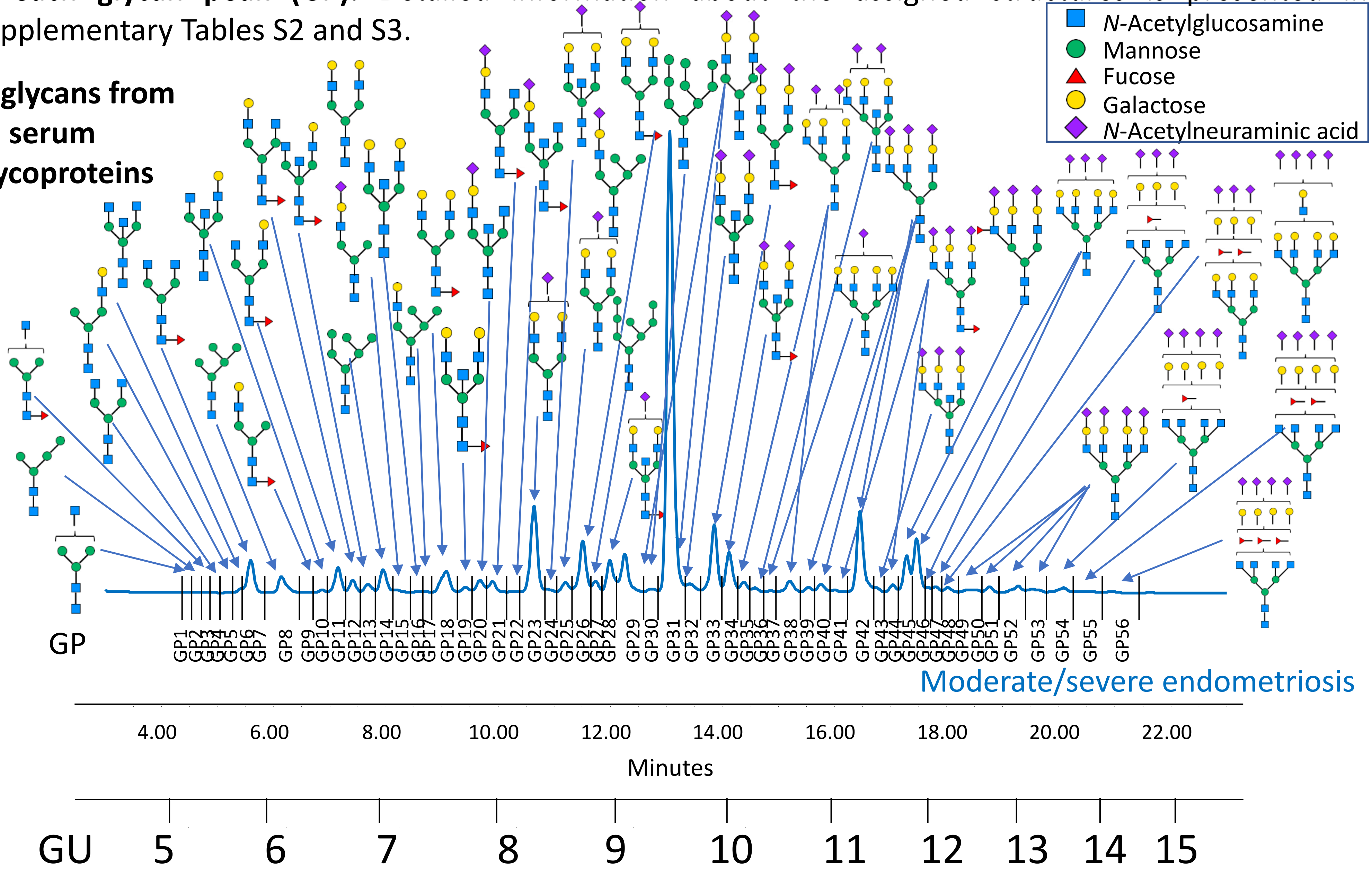

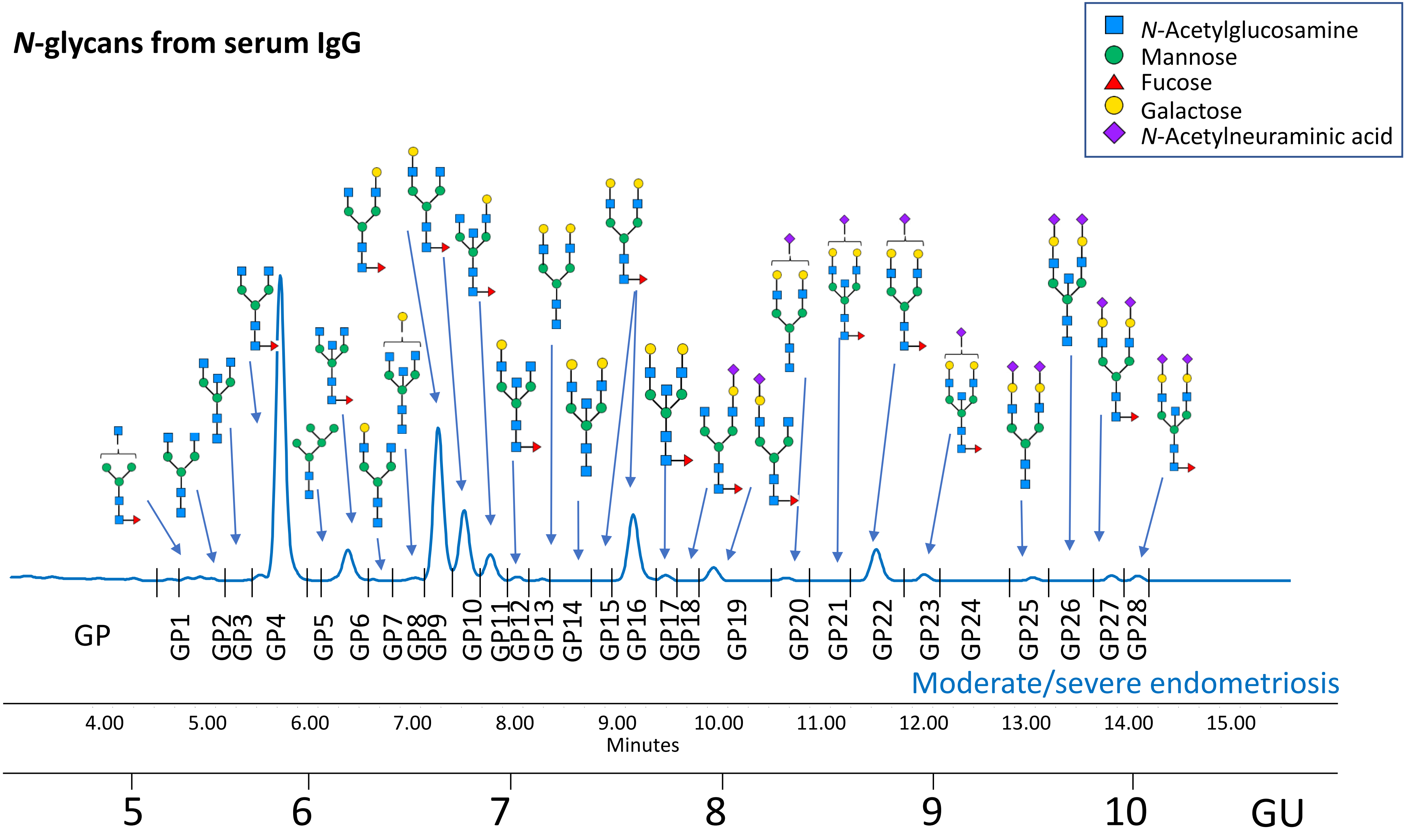
N-glycans from serum IgG
N-Acetylglucosamine
Mannose
Fucose
Galactose
N-Acetylneuraminic acid
GP
GP1 GP2 GP3 GP4 GP5 GP6 GP7 GP8 GP9 GP10 GP11 GP12 GP13 GP14 GP15 GP16 GP17 GP18 GP19 GP20 GP21 GP22 GP23 GP24 GP25 GP26 GP27 GP28
Moderate/severe endometriosis
4.00 5.00 6.00 7.00 8.00 9.00 10.00 11.00 12.00 13.00 14.00 15.00
Minutes
5 6 7 8 9 10
GU
