# Supplementary material for: N-glycans from serum IgG and total serum glycoproteins specific for endometriosis
Source: Sci Rep. 2023 Jun 28;13:10480. doi: 10.1038/s41598-023-37421-5 (PMC10307818; doi:10.1038/s41598-023-37421-5)

Supplementary Figure S2. GPs and features from *N*-glycans from whole serum and IgG glycoproteins which had at least one significant comparison among groups.

IgG GPx=serum IgG glycan peaks
Serum_GPx or feature=whole serum glycan peaks or features
Red star=significant change comparing to controls after Bonferroni correction for multiple testing

Whole serum GP11

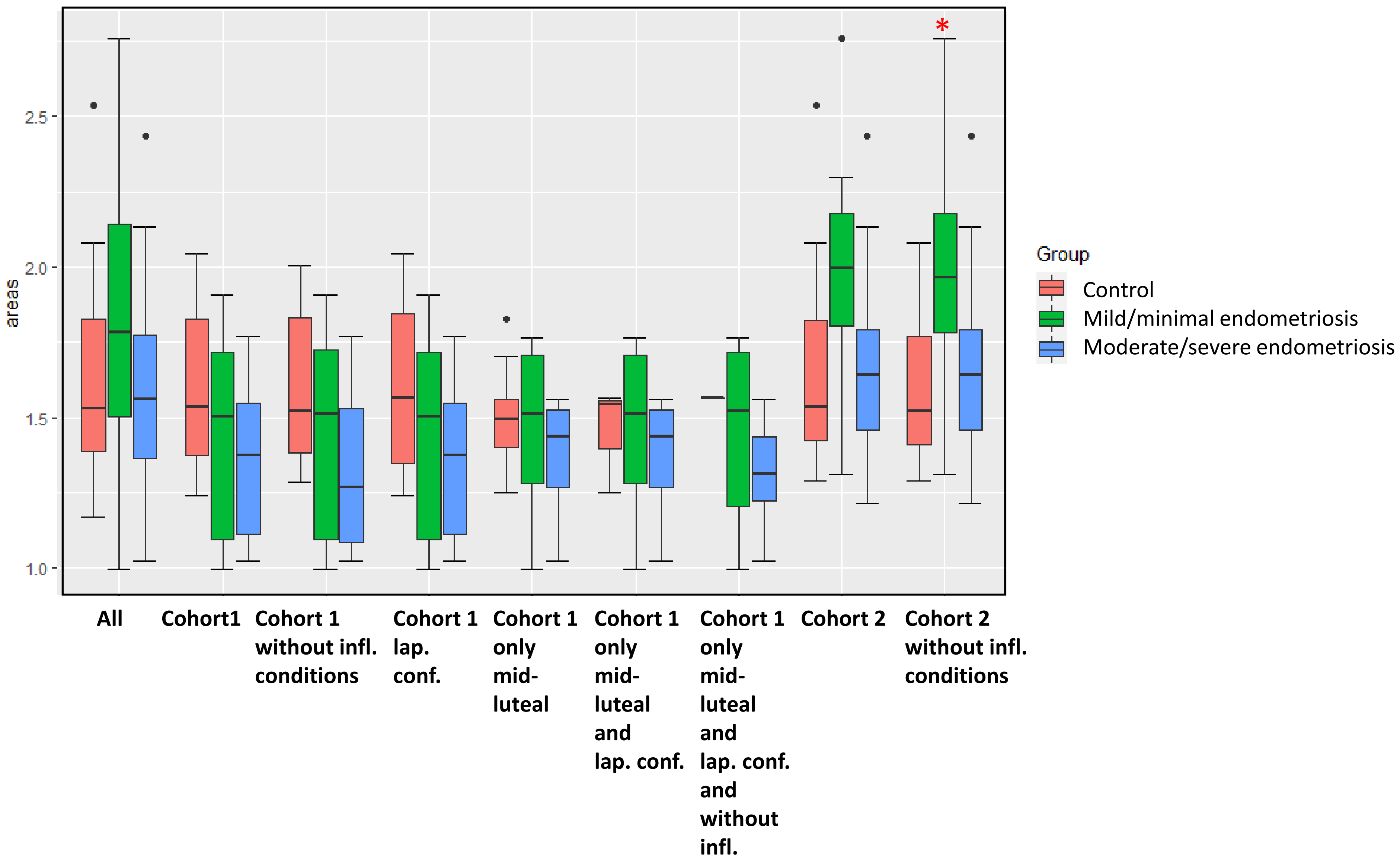

Whole serum GP15

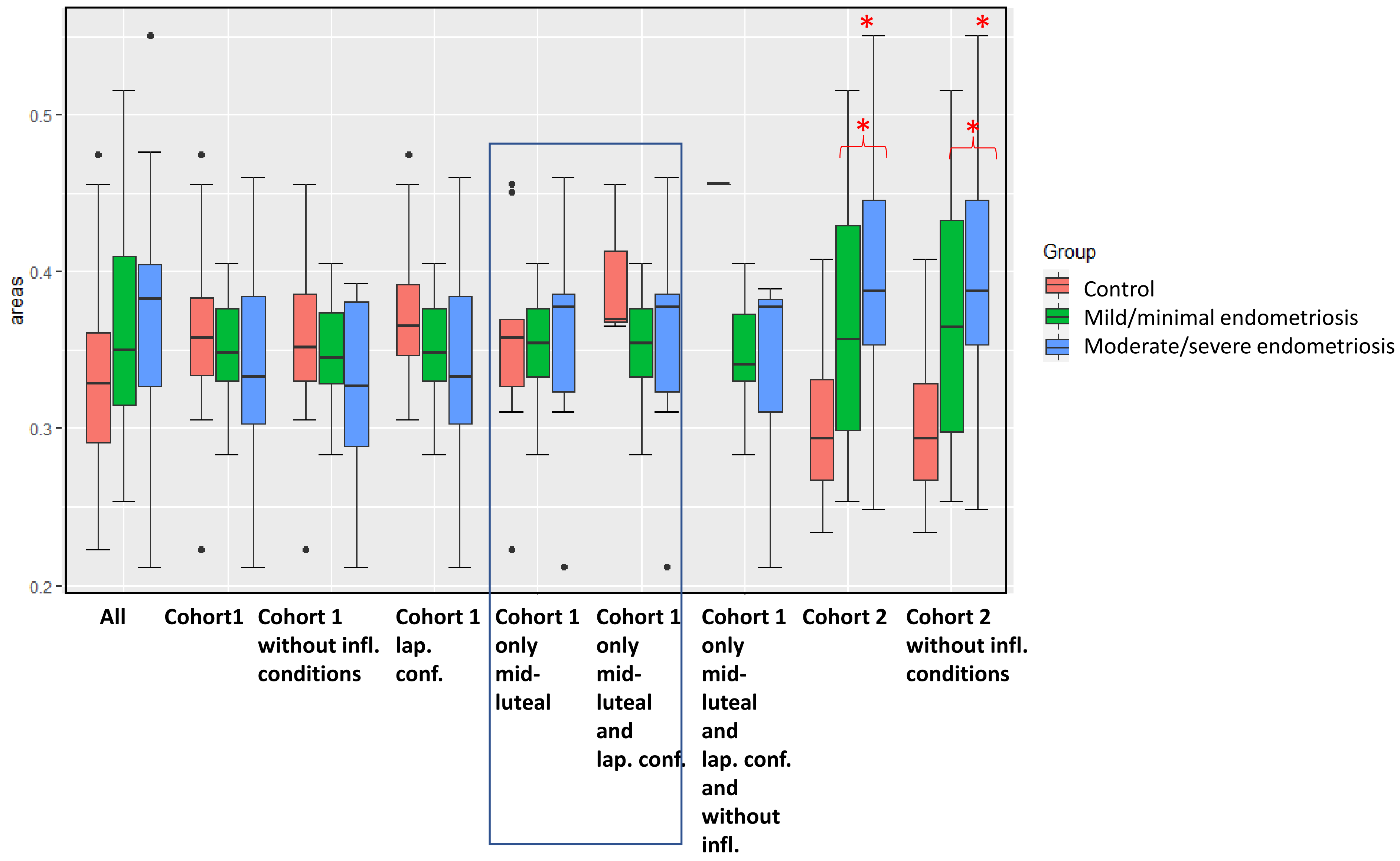

Whole serum GP22

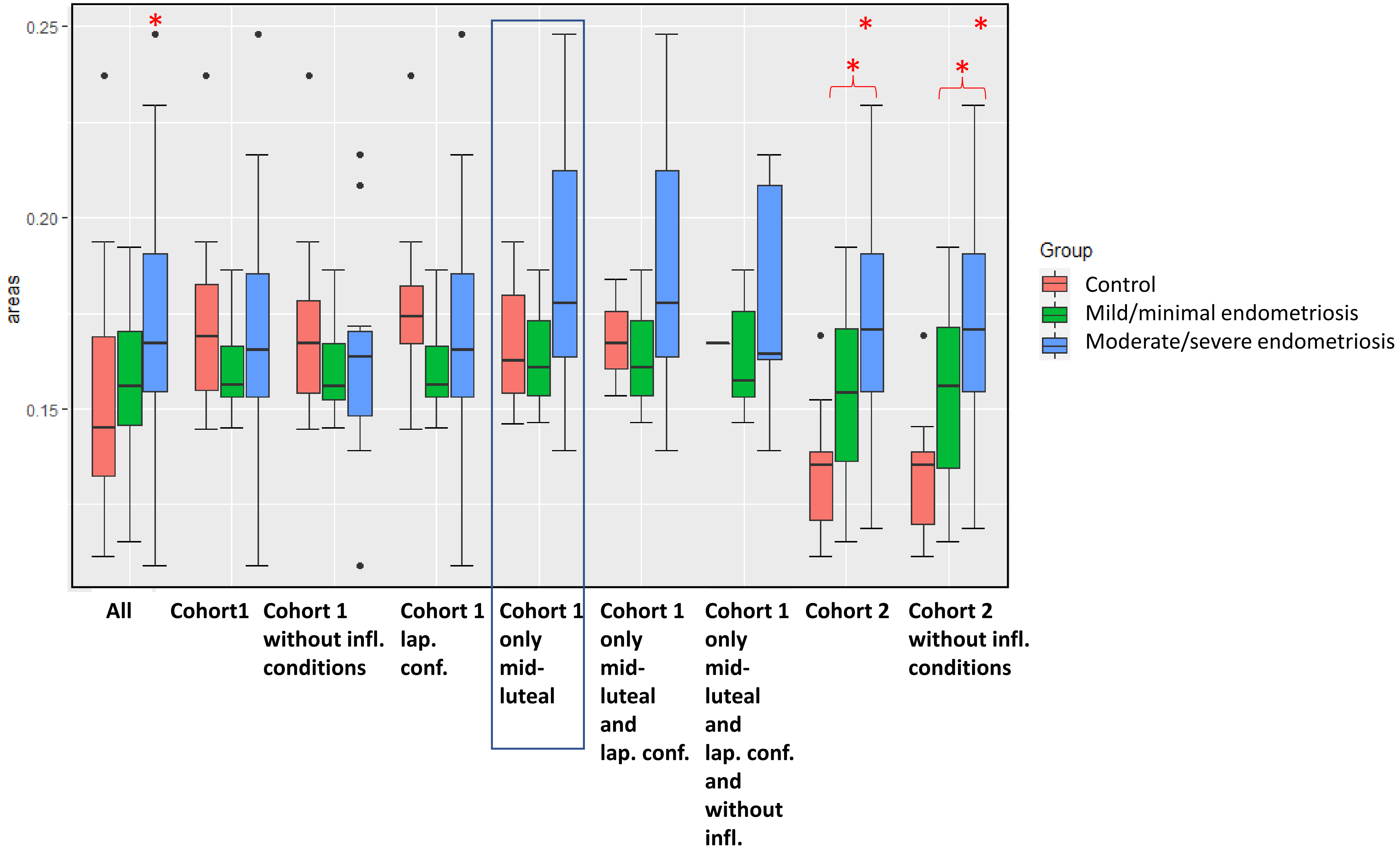

Whole serum GP24

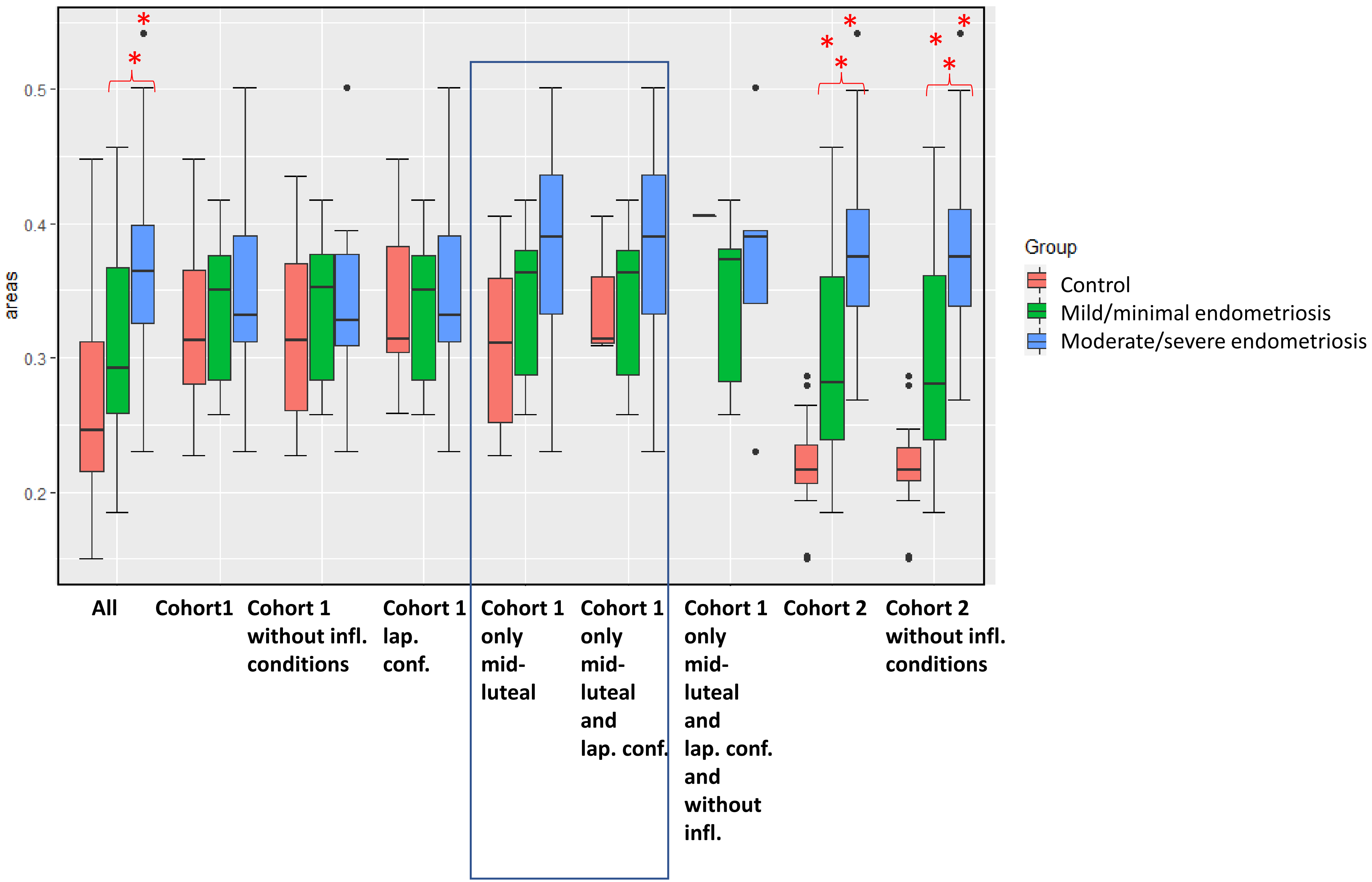

Whole serum GP44

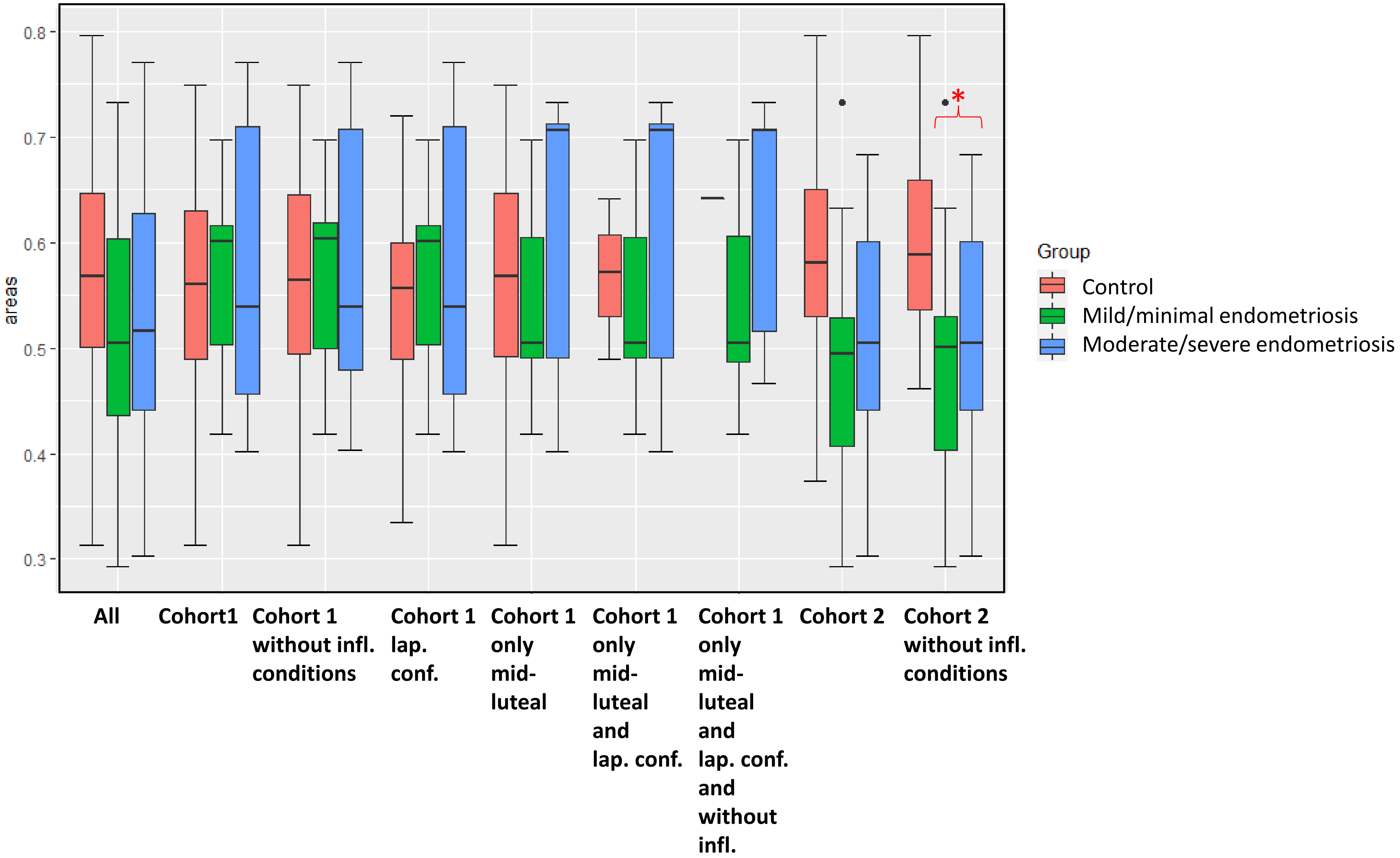

Whole serum GP50

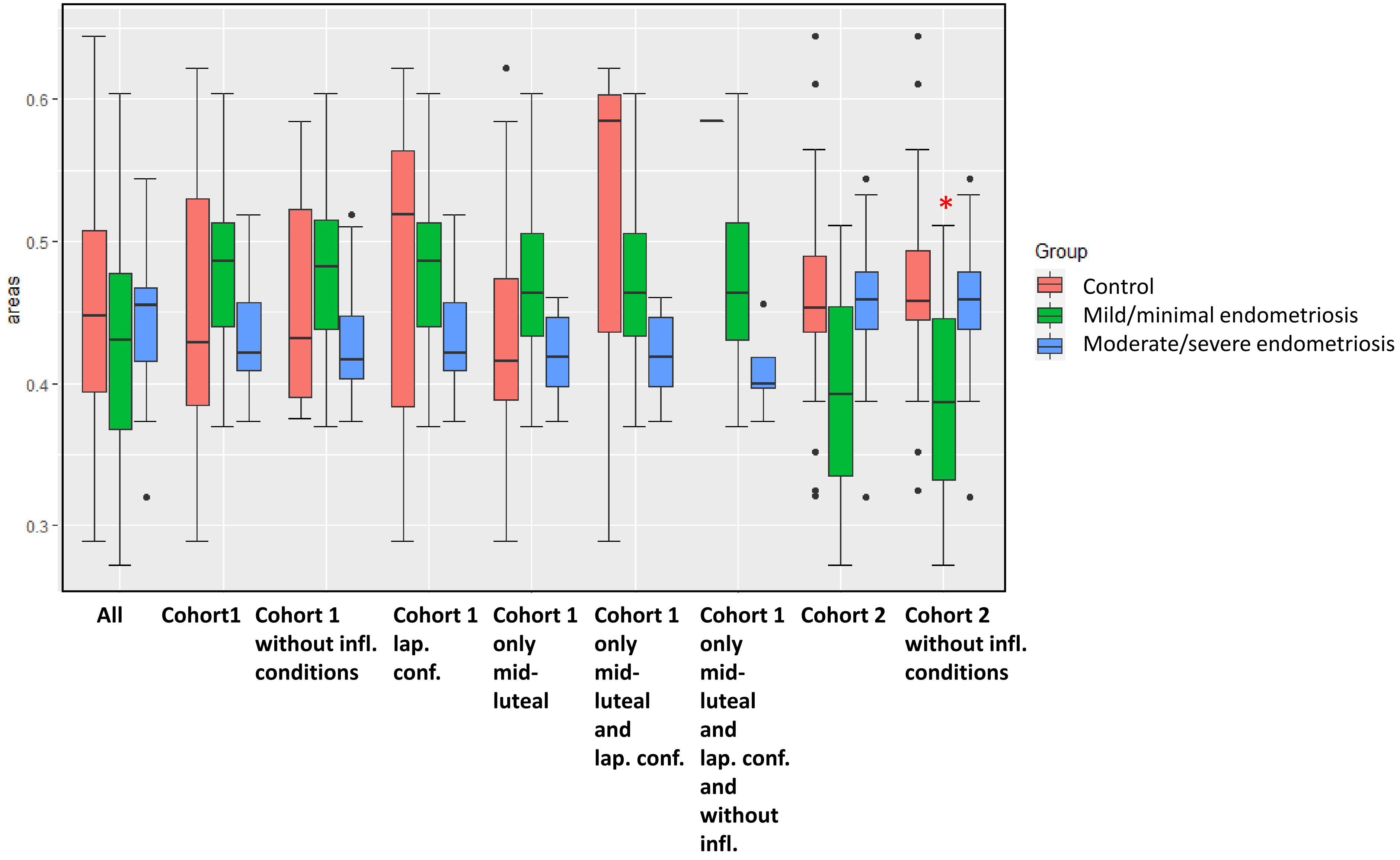

Whole serum GP52

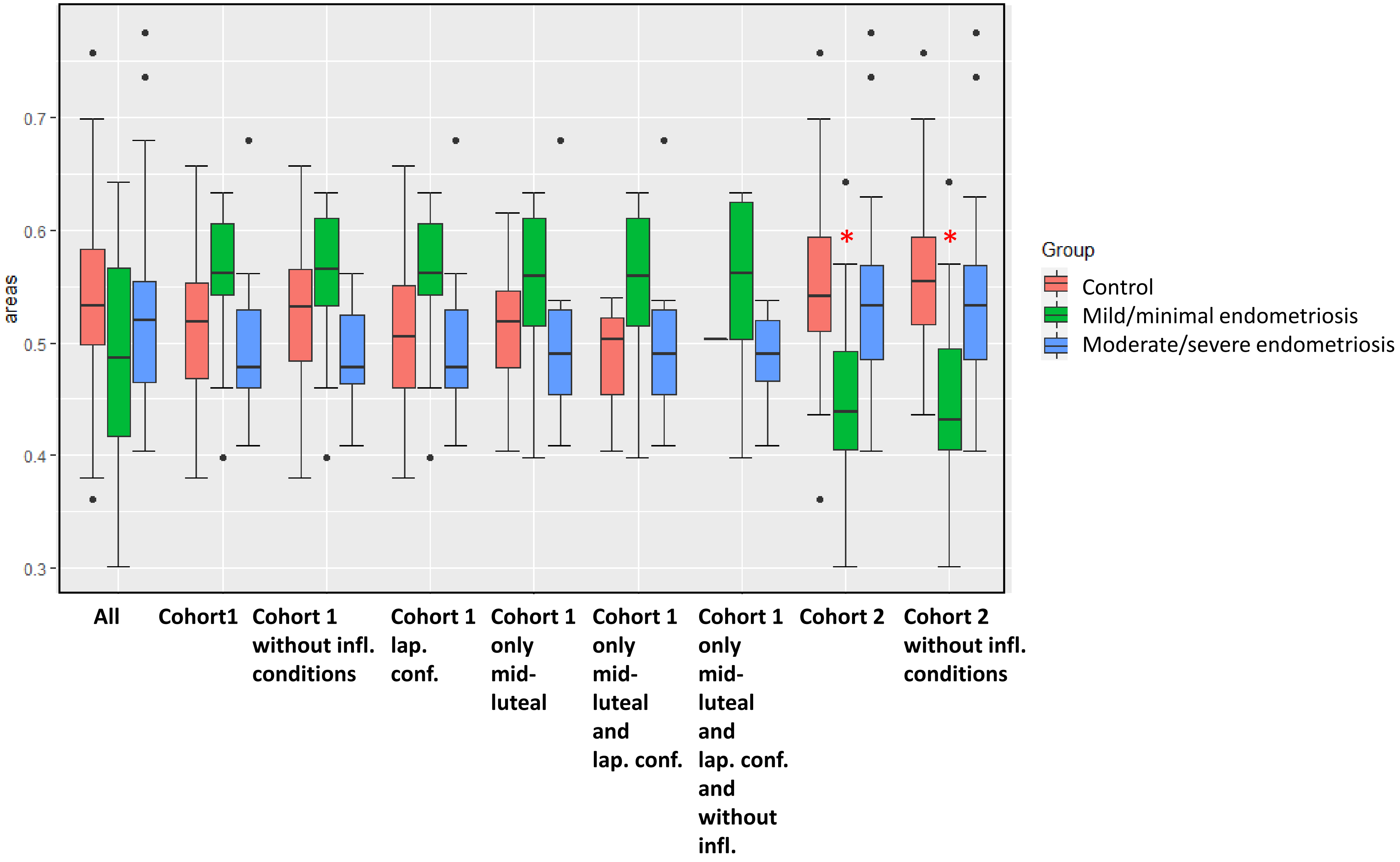

Whole serum GP53

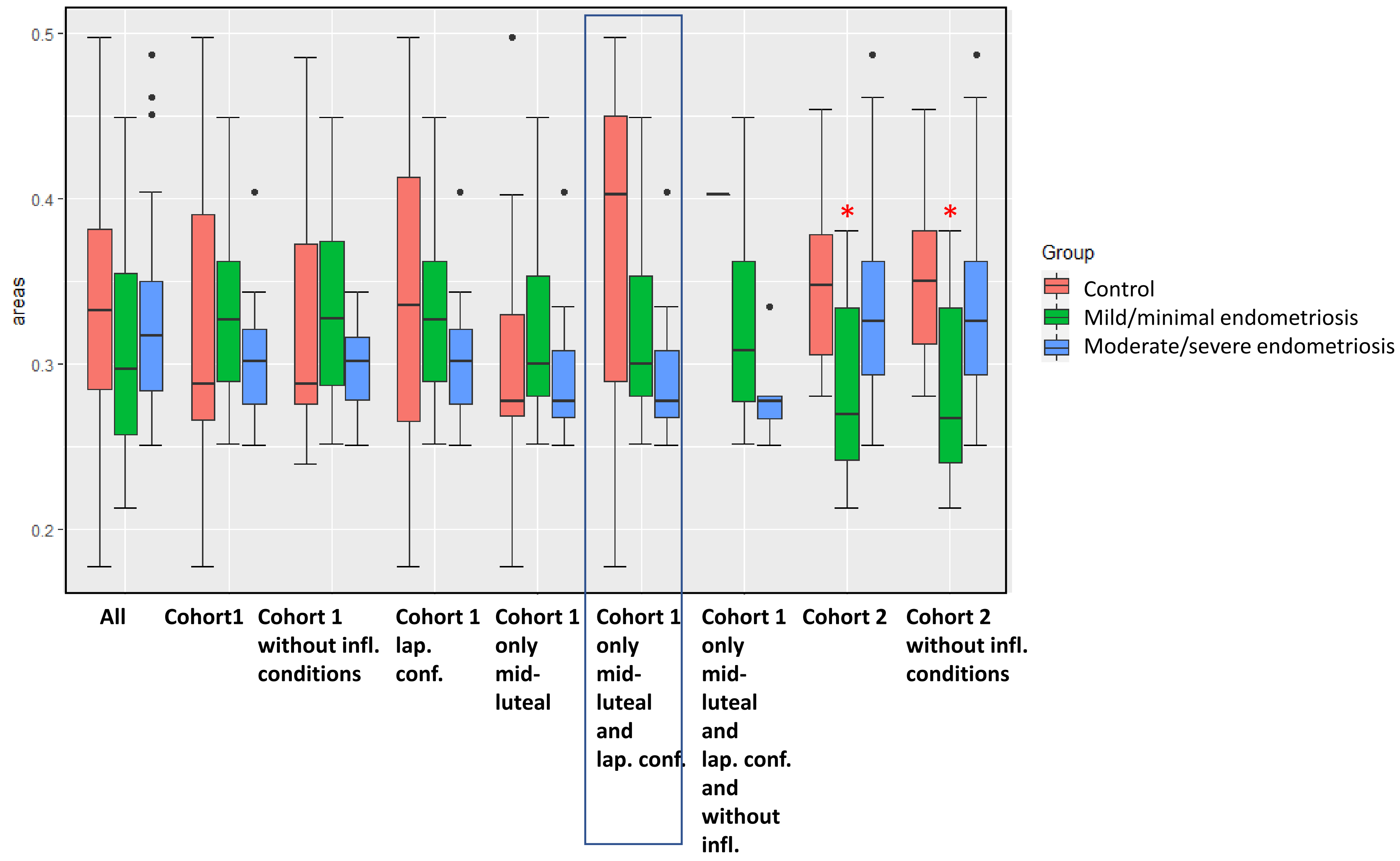

Whole serum S0

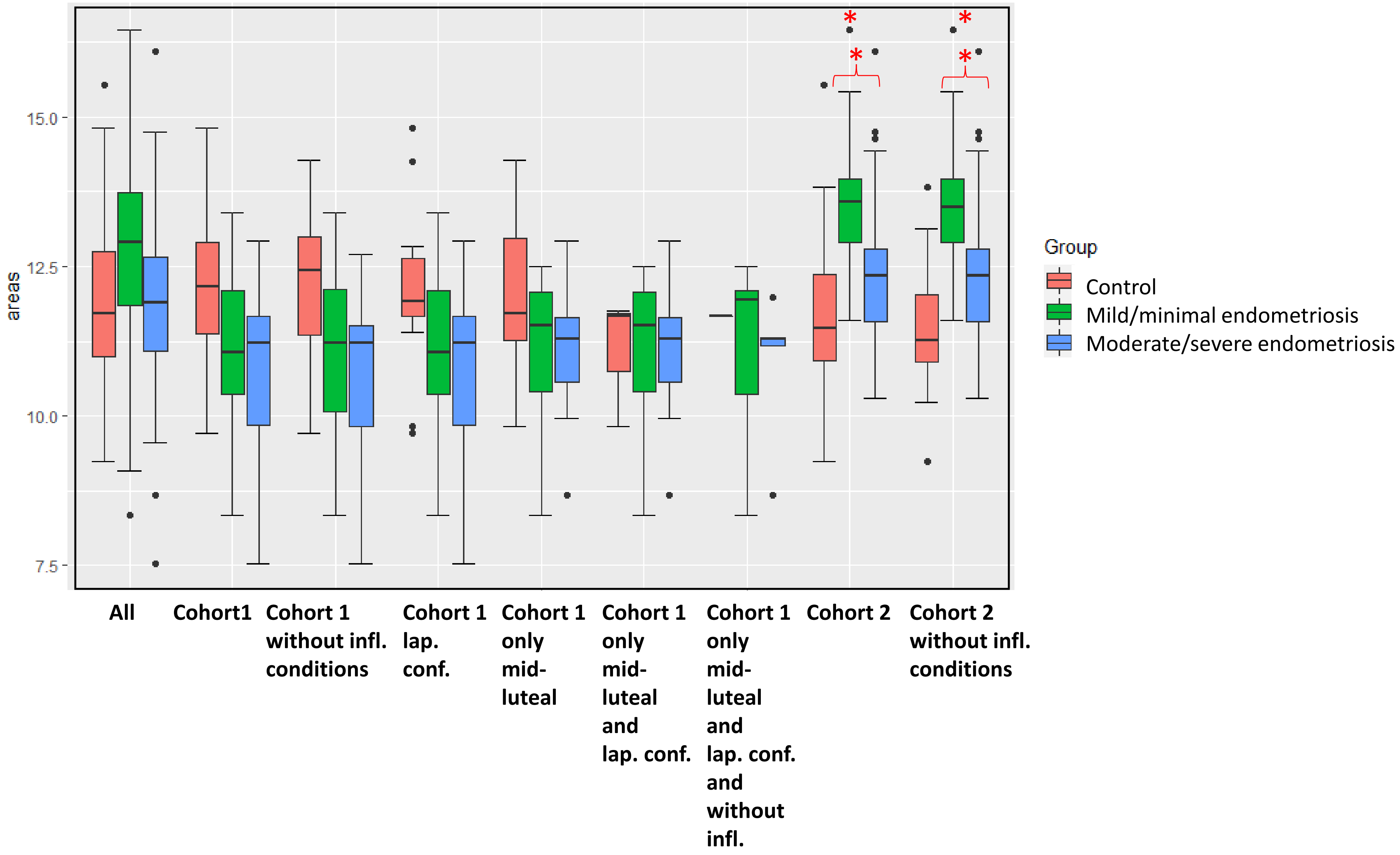

Whole serum S1

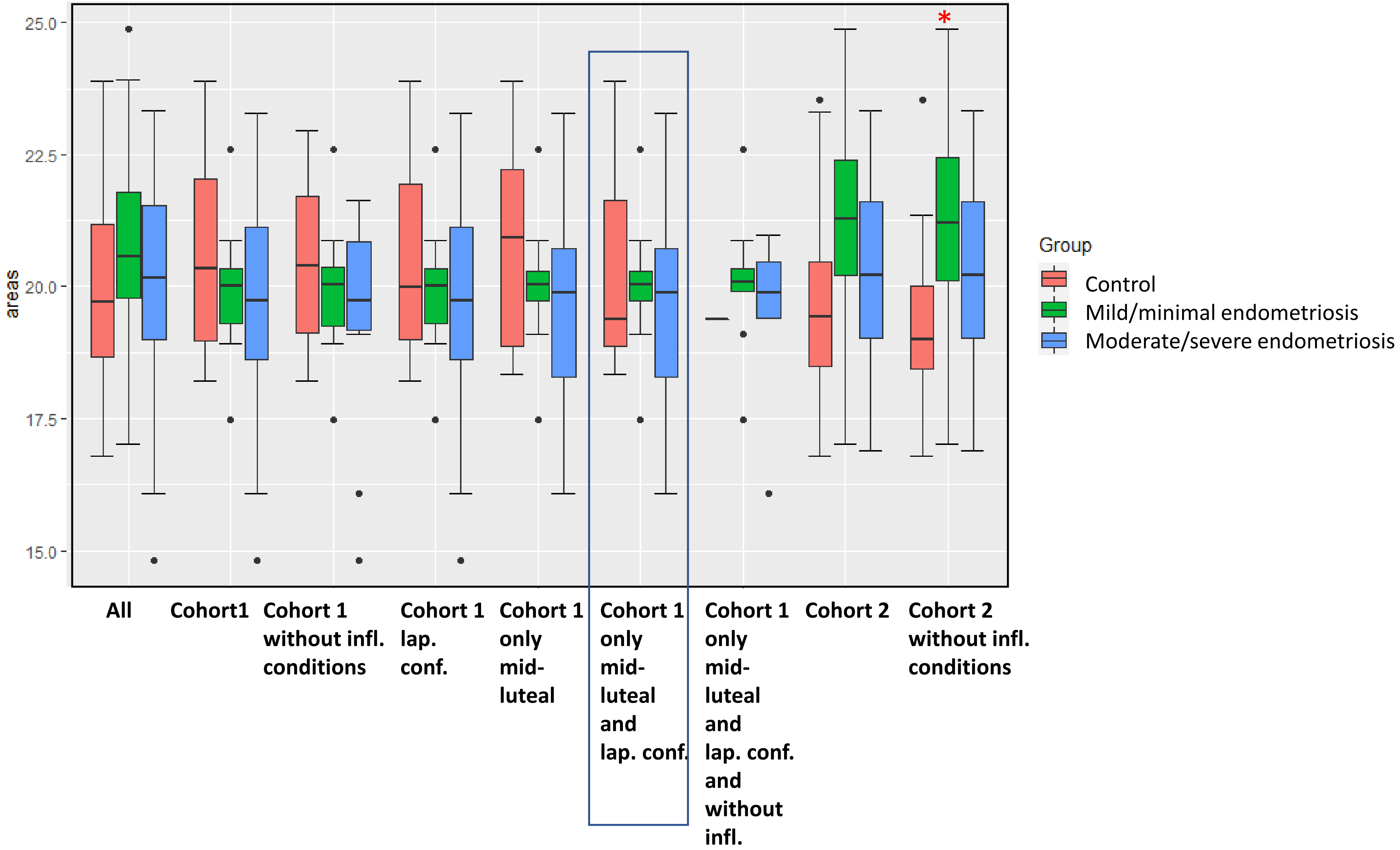

Whole serum S3

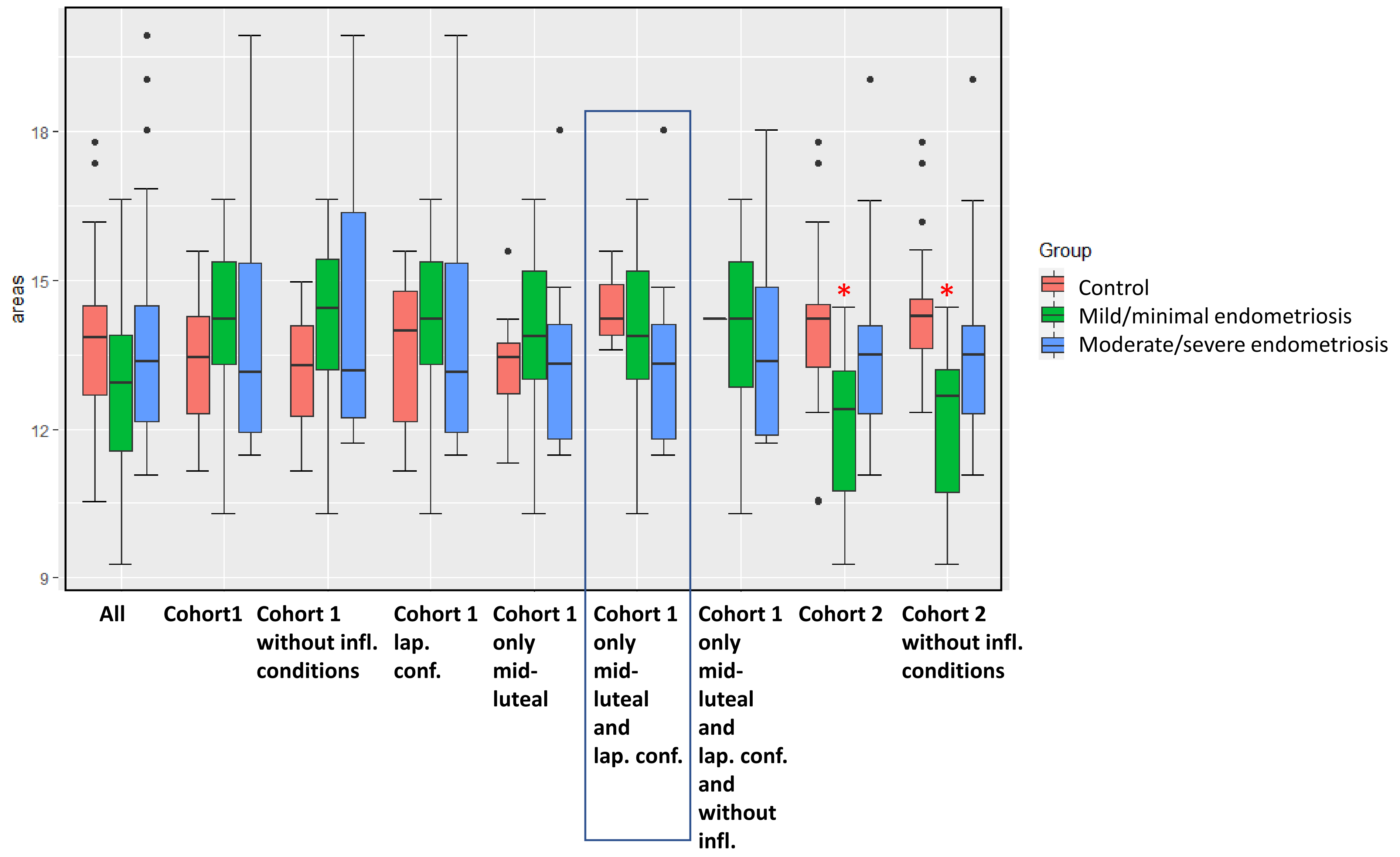

Whole serum S4

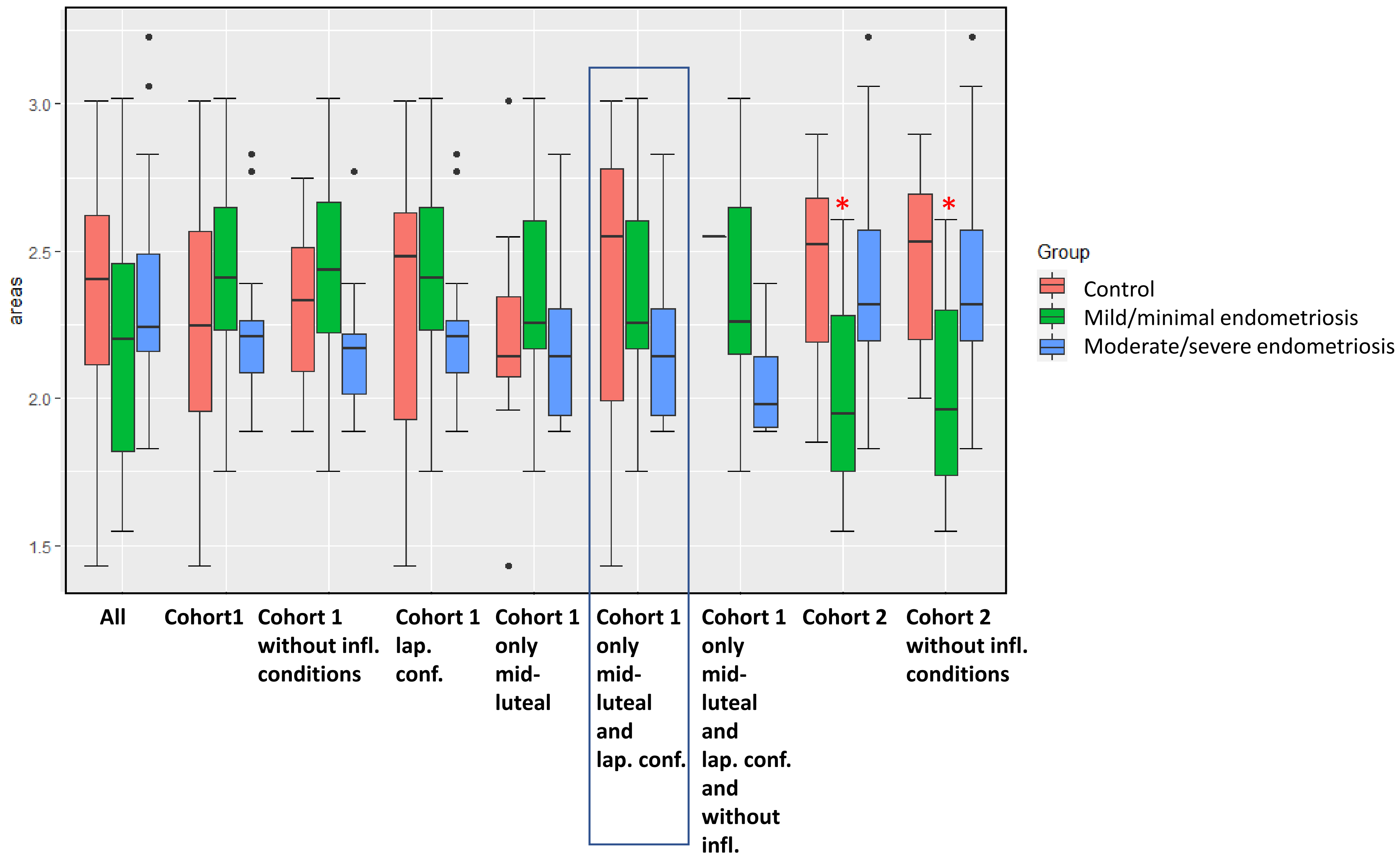

Whole serum G0

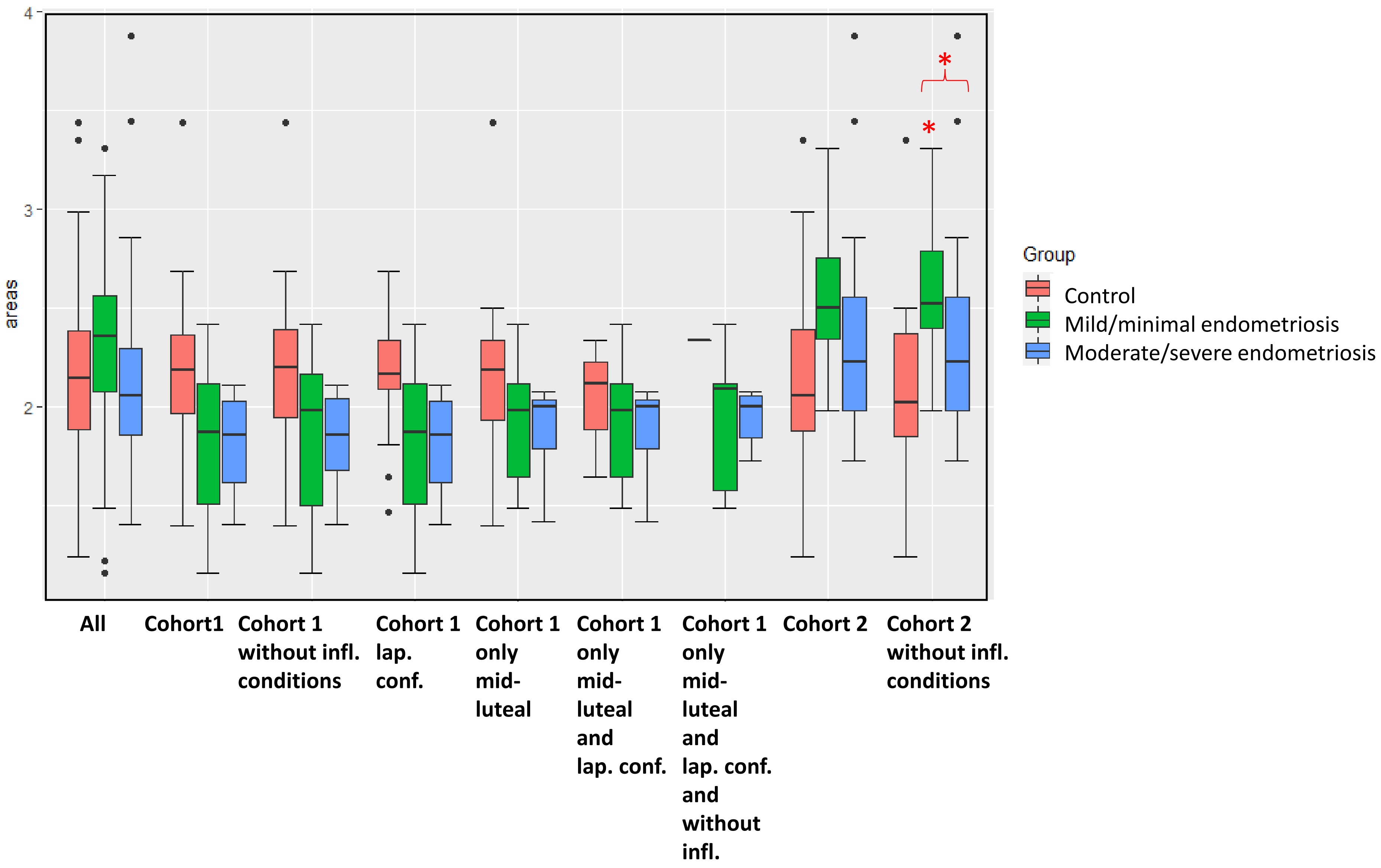

Whole serum G1

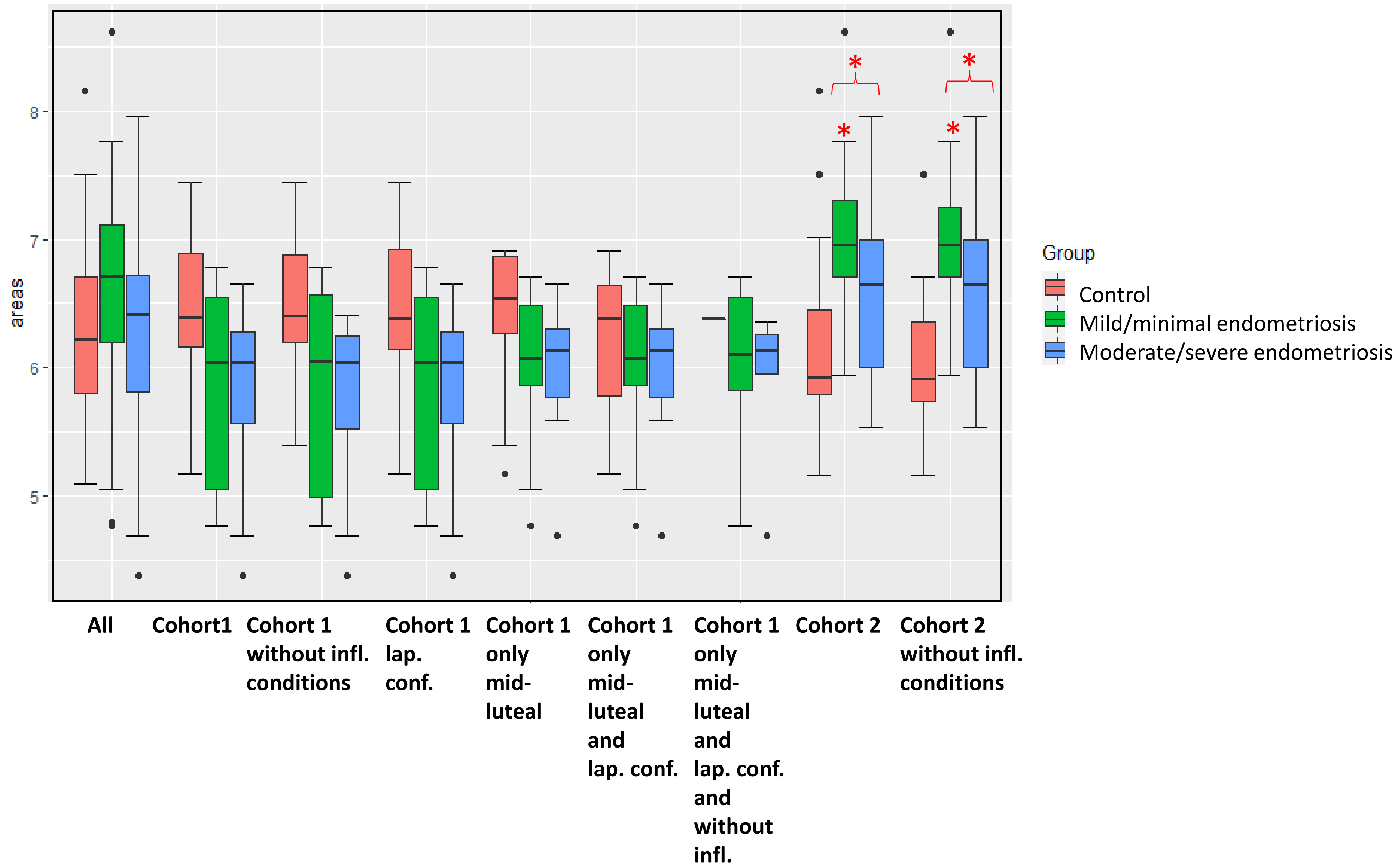

Whole serum G3

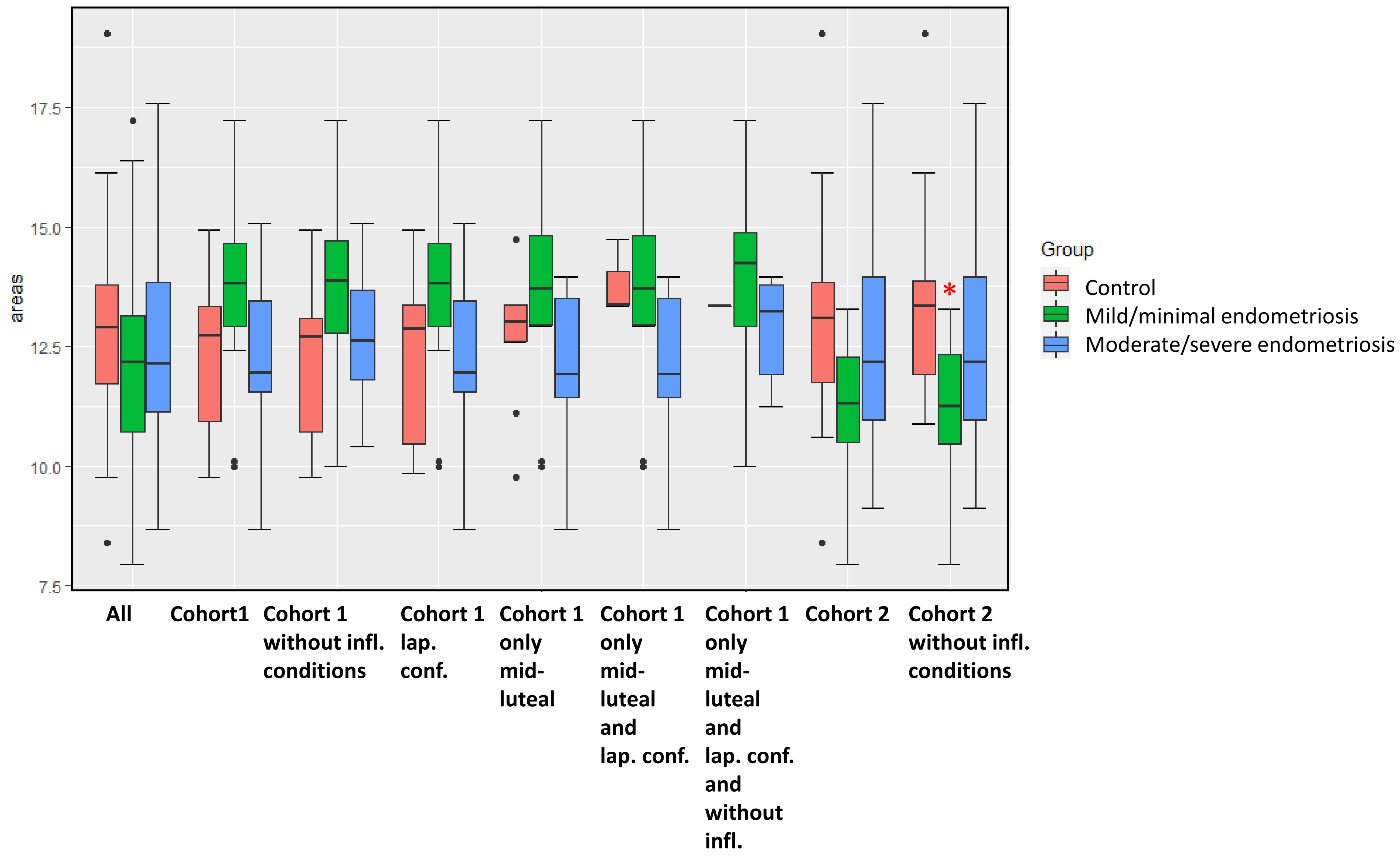

Whole serum A1

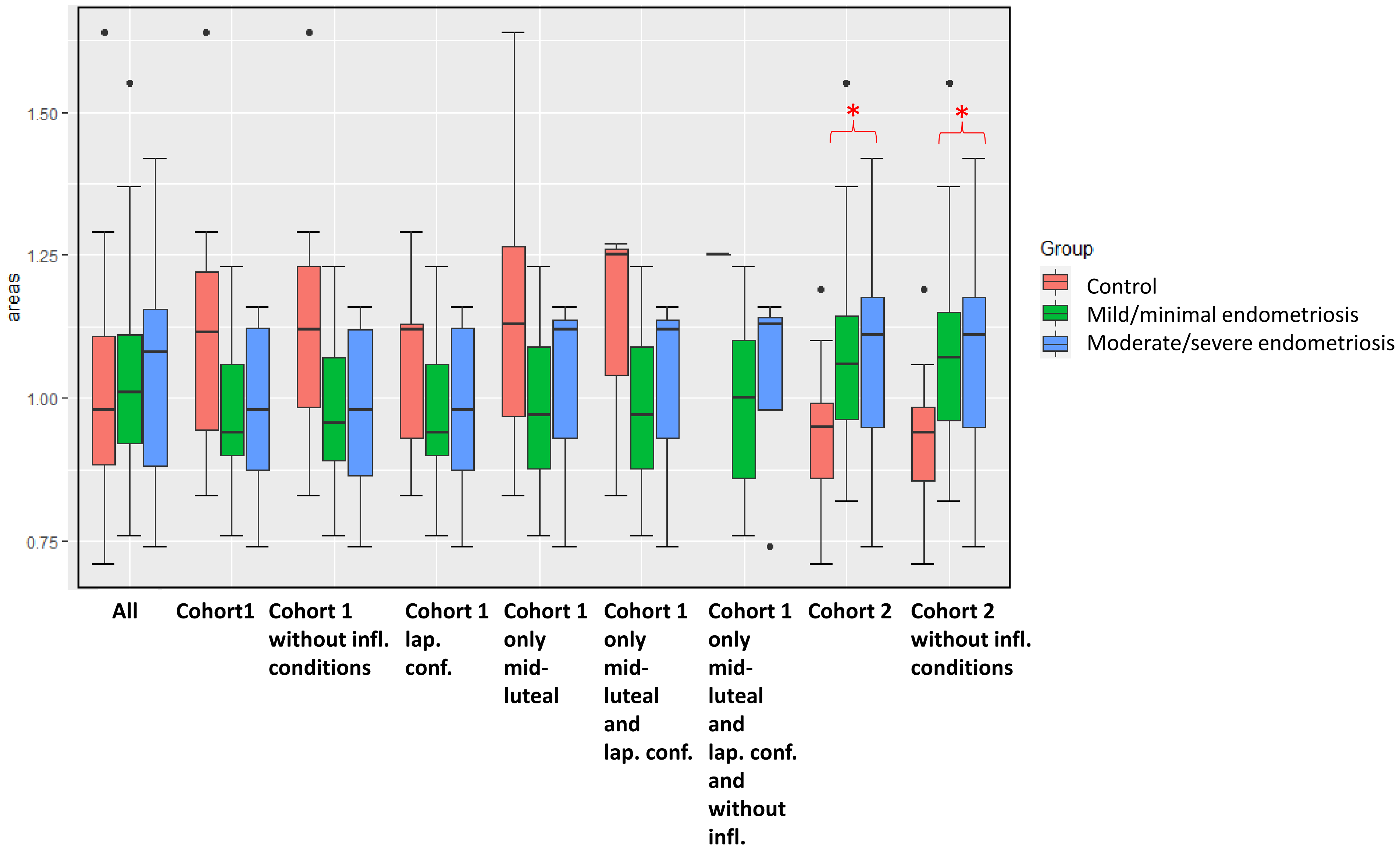

Whole serum A2

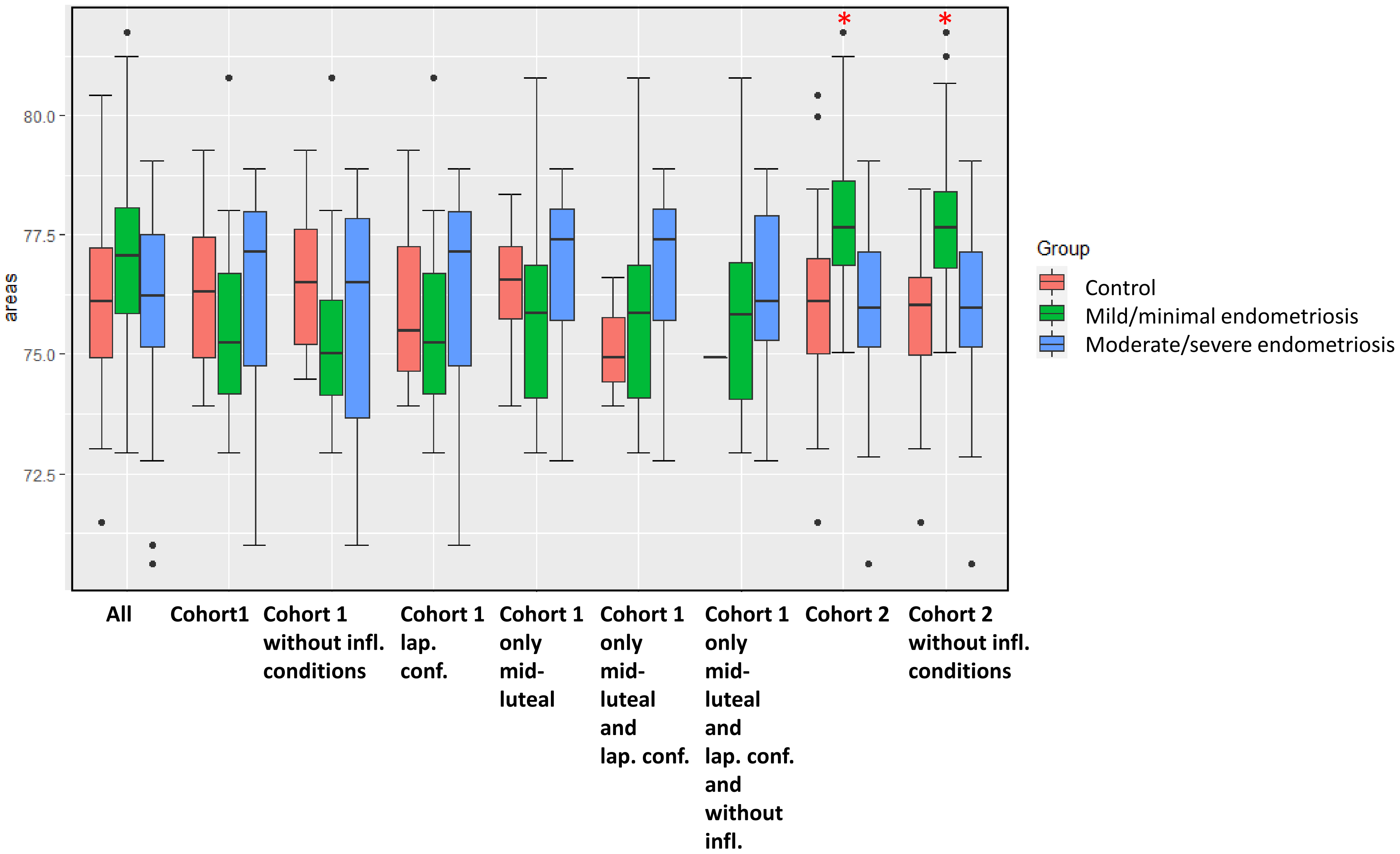

Whole serum A3

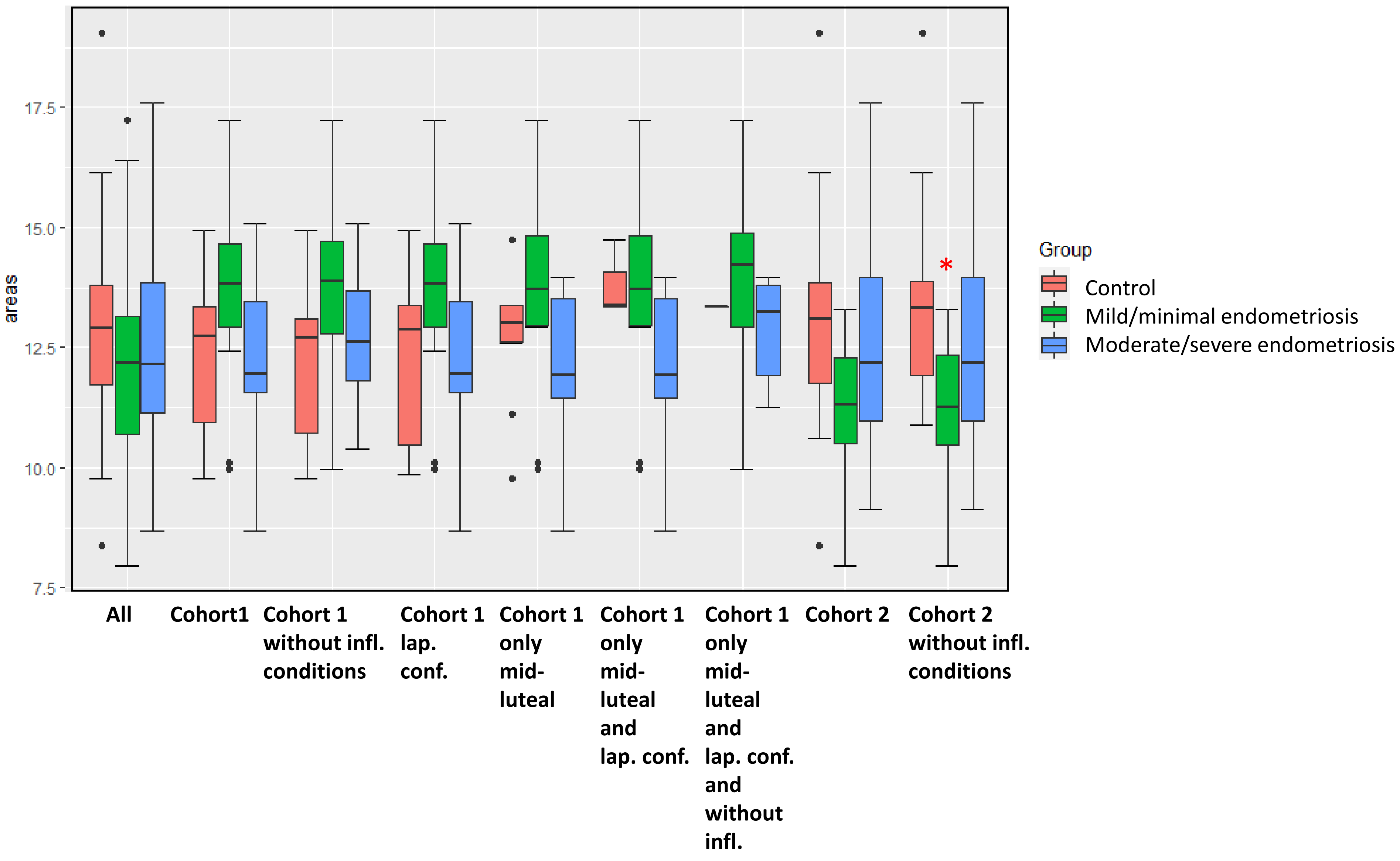

Whole serum outer arm fucose

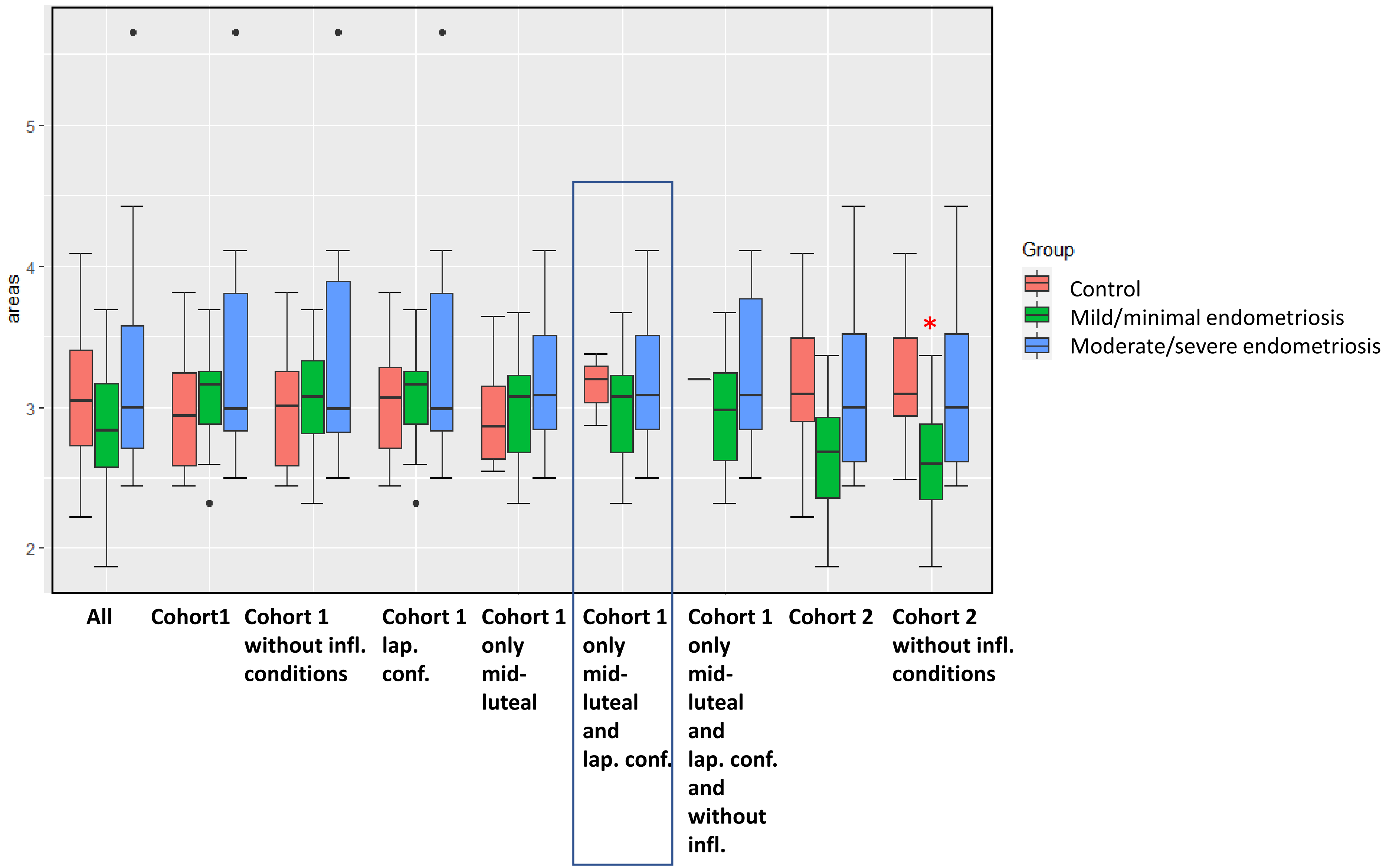

Serum IgG GP8

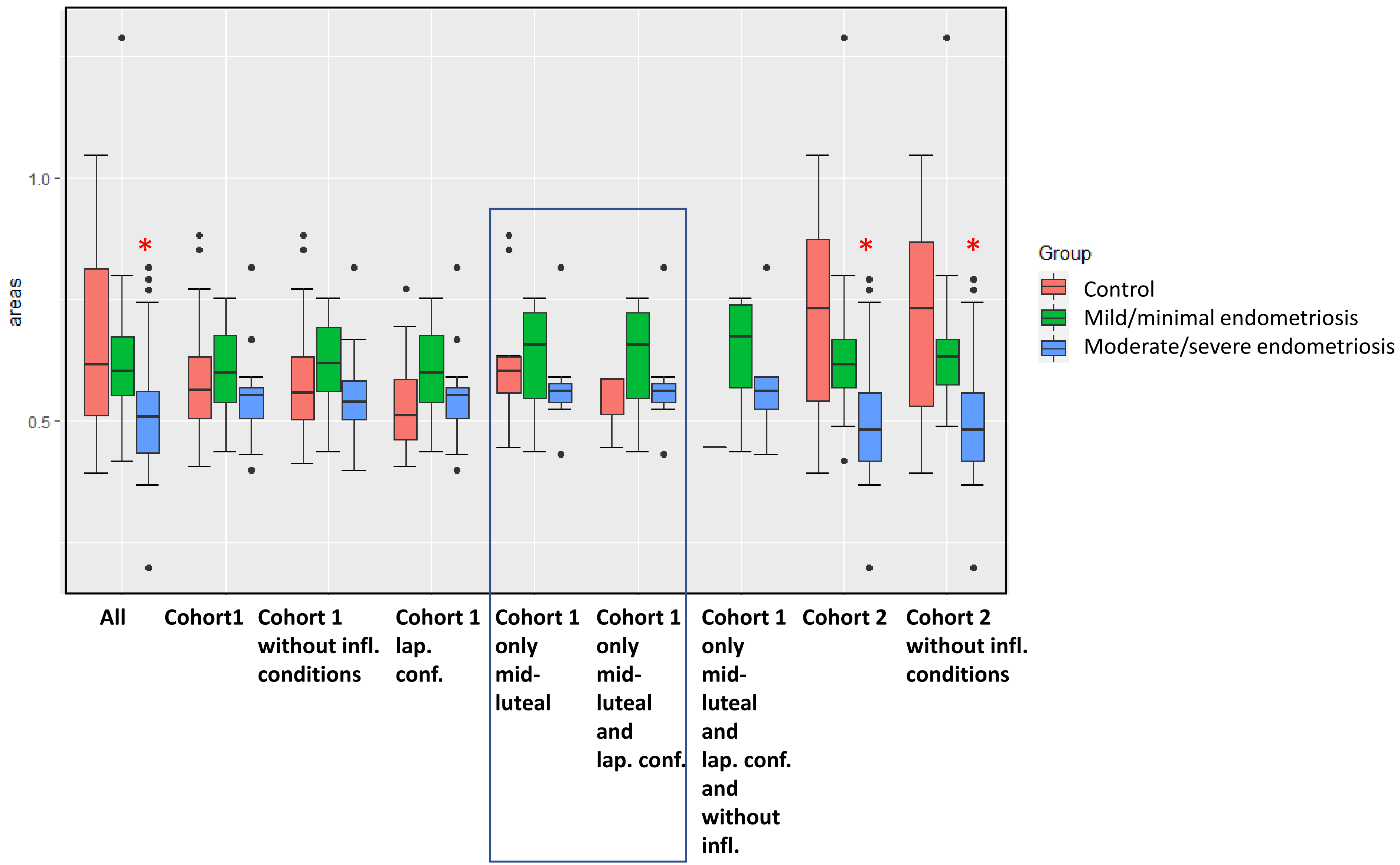

Supplement: Supplementary file 2 — Supplementary Figure S2. [file 41598_2023_37421_MOESM2_ESM.pdf]
