# Supplementary material for: N-glycans from serum IgG and total serum glycoproteins specific for endometriosis
Source: Sci Rep. 2023 Jun 28;13:10480. doi: 10.1038/s41598-023-37421-5 (PMC10307818; doi:10.1038/s41598-023-37421-5)
Supplement: Supplementary file 3 — Supplementary Figure S3. [file 41598_2023_37421_MOESM3_ESM.pdf]

Supplementary Figure S3. Clustering of all subjects based on *N*-glycome from whole serum glycoproteins.

Separation based on whole serum glycans

Serum GPs

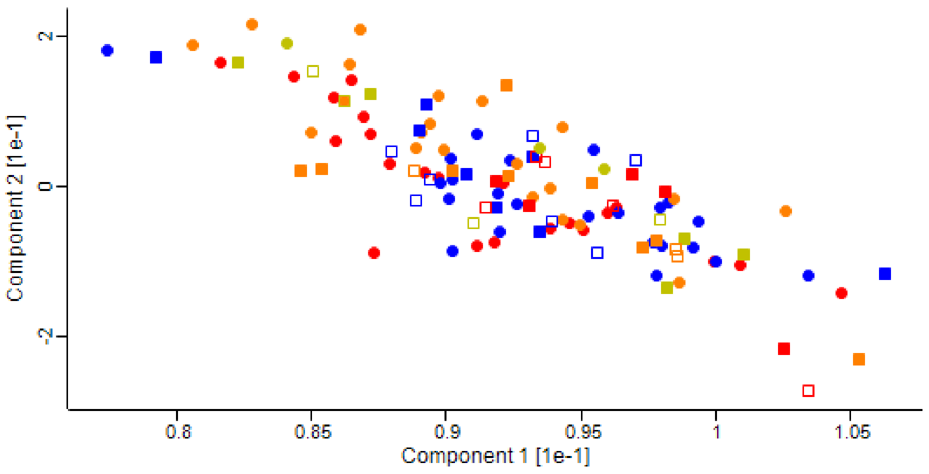

Serum features

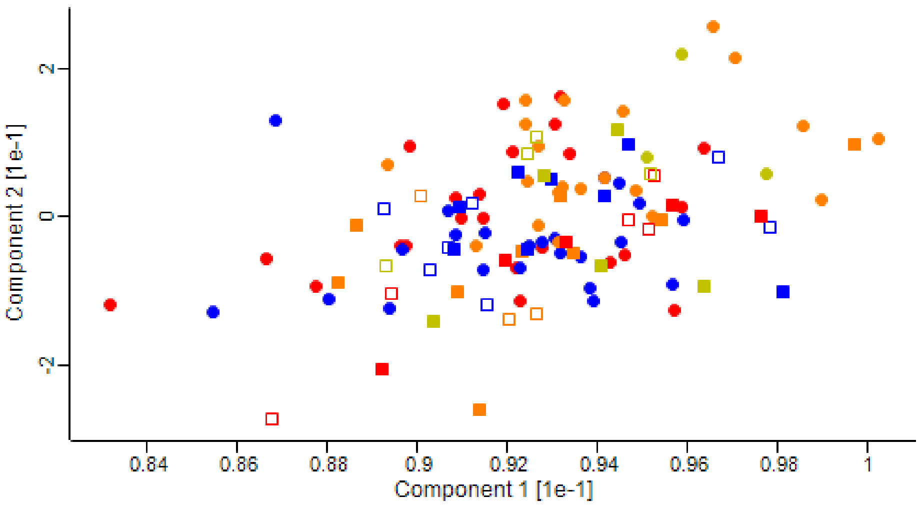

Cohort 1=square, filled square-mid-luteal phase only
Cohort 2=filled circle

Yellow- inflammatory samples

Blue=controls
Orange=mild/minimal endometriosis
Red=moderate/severe endometriosis
